# Self-Assembly of Soluplus in Aqueous Solutions: Characterization and Prospectives on Perfume Encapsulation

**DOI:** 10.1021/acsami.2c01087

**Published:** 2022-03-21

**Authors:** Constantina Sofroniou, Michele Baglioni, Marianna Mamusa, Claudio Resta, James Doutch, Johan Smets, Piero Baglioni

**Affiliations:** † Department of Chemistry “Ugo Schiff” and CSGI, University of Florence, Via della Lastruccia 3, Sesto Fiorentino, 50019 Florence, Italy; ‡ Science and Technology Facilities Council, ISIS Neutron and Muon Source, Rutherford Appleton Laboratory, Didcot OX11 0QX, United Kingdom; § The Procter & Gamble Company, Temselaan 100, 1853 Strombeek Bever, Belgium

**Keywords:** self-assembly, amphiphilic graft-polymer, fragrances, encapsulation, self-assembled capsules, small-angle neutron scattering

## Abstract

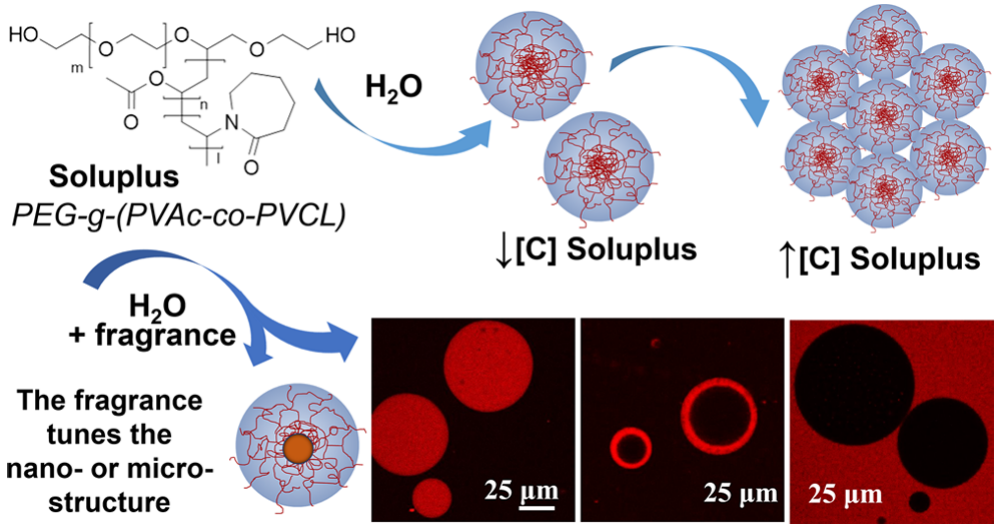

Soluplus is an amphiphilic graft copolymer intensively studied as a micellar solubilizer for drugs. An extensive characterization of the nanostructure of its colloidal aggregates is still lacking. Here, we provide insights into the polymer’s self-assembly in water, and we assess its use as an encapsulating agent for fragrances. The self-assembly properties of Soluplus aqueous solutions were studied over a wide concentration range (1–70% w/w) by means of small-angle neutron scattering (SANS), differential scanning calorimetry, NMR, and rheometry. SANS analyses revealed the presence of polymeric micelles with a fuzzy surface interacting via a 2-Yukawa potential, up to 15% w/w polymer. Increasing the polymer concentration up to 55% w/w led to tightly packed micelles described according to the Teubner–Strey model. The ability of Soluplus to encapsulate seven perfume molecules, 2-phenyl ethanol, l-carvone, linalool, florhydral, β-citronellol, α-pinene, and *R*-limonene, was then examined. We showed that the fragrance’s octanol/water partition coefficient (log *K*_ow_), widely used to characterize the solubilization capacity, is not sufficient to characterize such systems and the presence of specific functional groups or molecular conformation needs to be considered. In fact, the combination of SANS, NMR, confocal laser scanning microscopy, and confocal Raman microscopy showed that the perfumes, interacting with different regions of the polymer aggregates, are able to tune the systems’ structures resulting in micelles, matrix-type capsules, core–shell capsules, or oil-in-water emulsions.

## 1 Introduction

**Scheme 1:**
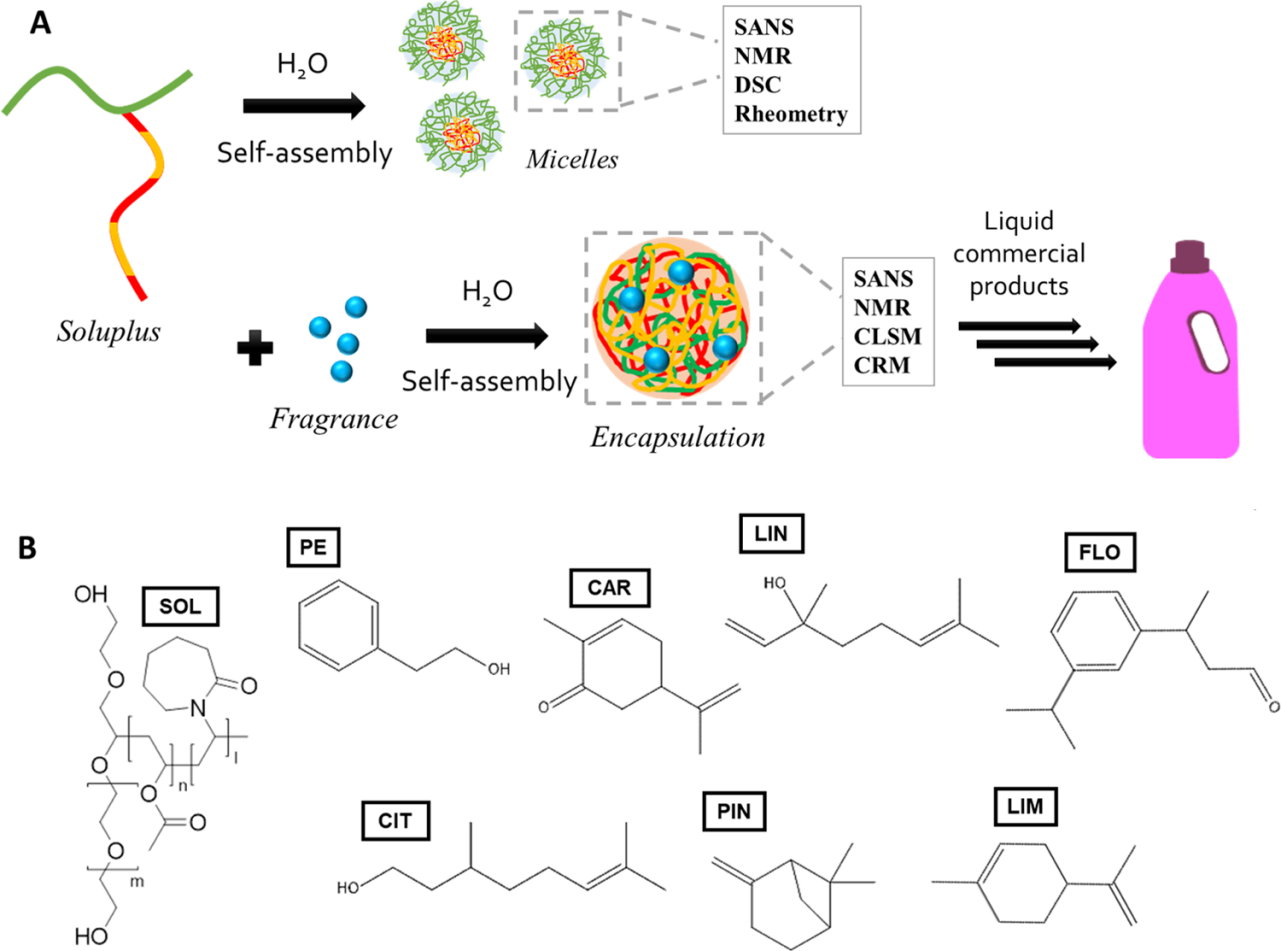
(A) Graphical Representation of the Design and Strategy of This Work. Soluplus’ Self-Assembly to Form Micellar Structures in Water Was Characterized Using Small-Angle Neutron Scattering (SANS), NMR Spectroscopy, Differential Scanning Calorimetry (DSC), and Rheometry. Once Known the Soluplus Self-Assembly Behavior, Its Encapsulation Properties for Seven Different Fragrances, with Potential Application in Consumers’ Liquid Formulations, Were Studied; (B) Molecular Structures of the Compounds Used in This Work SOL: Soluplus (PEG-*g*-(PVAc-*co*-PVCL)), PE: 2-phenyl ethanol, CAR: l-carvone, LIN: linalool, FLO: florhydral, CIT: β-citronellol, PIN: α-pinene, and LIM: *R*-limonene.

Amphiphilic block copolymers have been intensively employed in the last few decades, thanks to their ability to form a wide range of nanostructured systems,^1^ leading to an extremely rich application portfolio for drug delivery,^2^ home and personal care,^3,4^ agriculture,^5^ and other products. A great deal of attention has been drawn especially by amphiphilic copolymers showcasing a biocompatible and environmentally friendly profile, which have found applications in the pharmaceutical,^6^ food,^7^ and cosmetics industries.^8^ Soluplus, or poly(ethylene glycol)—poly(vinyl acetate)—poly(vinyl caprolactam) graft copolymer (PEG-*g*-(PVAc-co-PVCL)), (Scheme 1), is a nonlinear copolymer exhibiting a 6 kDa PEG backbone and a grafting chain composed of VAc and VCL units. Its biocompatible profile and amphiphilic nature have contributed to its wide usage as a solubilizing medium for several poorly water-soluble drugs (among others: quercetin,^9^ carvedilol,^10^ and lipoic acid^11^) and as a matrix former for the manufacture of solid dispersions.^12,13^

In a previous work, we have studied the phase behavior of an amphiphilic nonlinear copolymer of a similar structure, PEG-*g*-PVAc, in the presence of three common perfume raw materials (PRMs) (2-phenyl ethanol, l-carvone, and α-pinene) with different hydrophobicity, as expressed by the logarithm of the octanol/water partition coefficient, log *K*_ow_, but similar bulkiness.^14^ The polymer/PRM/water ternary systems were investigated in the 10–90% concentration range at 25 °C, to explore the effect of perfumes and their hydrophobicity on the phase behavior of the polymer. Several different structured systems were found in the 2-phenyl ethanol and l-carvone phase diagrams with PEG-*g*-PVAc and water, including single-chain nanoparticles, lamellar liquid crystals, and polymeric microcapsules, while α-pinene led to the formation of three-phase samples, likely due to its high hydrophobicity. Here, we are extending previous studies using seven different PRMs, which not only cover a wide hydrophobicity range but also exhibit a variety of functional groups and different molecular conformations. In the present work, we focus on diluted systems, which are most appealing for industrial applications, to explore the potential usage of Soluplus as an encapsulating agent for fragrances in commercial formulations. The significant presence (57% w/w) of VCL (a relatively hydrophilic monomer) in Soluplus’ graft chain greatly changes its hydrophilic/hydrophobic balance with respect to PEG-*g*-PVAc, also favoring its interaction with more hydrophilic molecules. On the other hand, Soluplus graft chain is sensibly longer than PEG-*g*-PVAc’s, and in principle, this confers to Soluplus a higher degree of conformational freedom to interact with perfume molecules. This makes Soluplus a strong candidate for the encapsulation of chemicals spanning a wide range of hydrophobicity. Since perfume accords used in homecare or cosmetics industry can be composed of dozens to hundreds of PRMs and essential oils having different hydrophobicities and molecular structures, a polymer able to interact with molecules within an ample range of hydrophobicity is needed. It is noteworthy to mention the tendency of the market in recent years, to move not only toward more environmentally friendly technologies for perfume encapsulation^15^ but also toward “smart” materials where the release of active can be triggered by external stimuli.^16−18^ In this sense, Soluplus is interesting because, besides the fact that the presence of VCL can potentially improve its biodegradability profile,^19,20^ it is also thermoresponsive^21,22^ possibly due to the fact that PVCL is thermosensitive, exhibiting a lower critical solution temperature (LCST) of 30–34 °C. Soluplus at low concentrations in water is known to self-assemble into micellar structures and has a critical micelle concentration (CMC) of 7.6 mg/L.^1^ Such micelles are reported to have an average hydrodynamic diameter (*D*_h_) of 55–65 nm, depending on polymer’s concentration and temperature.^23−26^ However, in-depth characterization concerning their structure, shape, and micelles interactions is still lacking. Increasing polymer’s concentration up to 50% w/w leads to a progressive increment of the viscosity and of the elastic properties of the material. Despite the gel-like appearance, no rheological evidence of network formation is reported.^10,13,26^ Therefore, the nanoscale structures and interactions characterizing these systems, both in diluted and concentrated solutions, merit investigation.

In the present work, Soluplus’ structure was first characterized through NMR, and its self-assembly properties in aqueous solutions were investigated by means of small-angle neutron scattering (SANS), rheometry, and differential scanning calorimetry (DSC). Then, the capability of Soluplus as an encapsulating agent for different model perfume molecules in aqueous solutions was assessed. The strategy followed in this work is graphically represented in Scheme 1A. Seven different fragrances with not only different octanol/water partition coefficients (log *K*_ow_) but also different functional groups and molecular conformation/bulkiness were used: 2-phenyl ethanol (PE), l-carvone (CAR), linalool (LIN), florhydral (FLO), β-citronellol (CIT), α-pinene (PIN) and *R*-limonene (LIM), whose molecular structures are reported in Scheme 1B. We report the phase behavior of Soloplus for a wide range of concentration, and we show that the log* K*_ow_ parameter is not sufficient to properly account for the solubilization properties of perfume molecules.

## 2 Materials and Methods

### 2.1 Materials

Soluplus (PEG-*g*-(PVAc-co-PVCL)) is a BASF product. The polymer is characterized by a PEG/PVAc/PVCL weight ratio of 13/34/53, range of molecular weight 90–140 kDa.^1^ For confocal microscopy experiments, Soluplus was covalently labeled with Rhodamine-B isothiocyanate, according to a procedure described elsewhere.^3^

The following reagents were used as received: 2-phenyl ethanol (Sigma-Aldrich, ≥99.0% (GC), log *K*_ow_ = 1.36, *M*_w_ 122.16 g mol^–1^), l-carvone (Sigma-Aldrich, ≥97%, (FCC, FG), log *K*_ow_ = 2.74, *M*_w_ 150.22 g mol^–1^), linalool (Symrise, ≥97%, (FCC, FG), log *K*_ow_ = 2.97, *M*_w_ 154.25 g mol^–1^), florhydral (Givaudan, ≥98%, log *K*_ow_ = 3.02, *M*_w_ 190.29 g mol^–1^), β-citronellol (Sigma-Aldrich, ≥95%, (FCC,FG), log *K*_ow_ = 3.30, *M*_w_ 156.27 g mol^–1^), α-pinene (Sigma-Aldrich, ≥99.0%, log *K*_ow_ = 4.44, *M*_w_ 136.23 g mol^–1^), *R*-limonene (Symrise, ≥95%, log *K*_ow_ = 4.57, *M*_w_ 166.26 g mol^–1^), and rhodamine-B isothiocyanate (mixed isomers, Sigma-Aldrich, *M*_w_ 536.08 g mol^–1^). Samples for SANS were prepared in D_2_O (deuterium content > 99%); H_2_O used in the rest of this work was Milli-Q grade (18.2 MΩ cm at 25 °C).

### 2.2 Samples Preparation

Samples with polymer concentrations ranging between 1 and 80% w/w in water were prepared to study the phase behavior of binary mixtures. The appropriate amounts of polymer and water were mixed, and samples were placed in an orbital shaker until homogenization and stabilized at 25 °C for 14 days. In the same way, for the preparation of samples containing PRMs, 50 mg of polymer were mixed with 940 mg of water until fully dissolved. Then, 10 mg of one of the PRMs were added and the solution was vortexed for a few seconds and stored at 25 °C. In this work, concentrations will always be expressed as weight percent (unless specified differently).

### 2.3 Nuclear Magnetic Resonance (NMR)

^1^H NMR and two-dimensional heteronuclear single-quantum coherence ({^1^H–^13^C}-HSQC), Nuclear Overhauser effect spectroscopy ({^1^H–^1^H}-NOESY), and correlated spectroscopy ({^1^H–^1^H}-COSY) experiments were performed by means of a Bruker AVANCE spectrometer operating at 400 MHz (^1^H) using the peak of the residual protonated solvent as an internal reference. Samples of Soluplus (without PRM addition) were prepared in DMSO-*d*_6_.^1^H NMR spectra of 2-phenyl ethanol was acquired in CDCl_3_.^1^H–^1^H-NOESY of the sample with 5% Soluplus and 1% 2-phenyl ethanol was acquired in D_2_O. NOESY experiments were conducted with mixing times of 200 and 500 ms, 512 experiments in the F1 dimension with 16 scans for each of the increments on t_1_, and a sweep width of 15 ppm.

### 2.4 Tensiometry

Measurement of the surface tension of Soluplus aqueous solutions was done with a KSV Sigma 70 static tensiometer (accuracy 0.1 mN/m), allowing an automatic determination of the CMC using the duNouy ring. The temperature was constant at 25.0 ± 0.5 °C in a controlled temperature vessel. CMC was measured by water dilution of a concentrated polymer solution.

### 2.5 Small-Angle Neutron Scattering (SANS)

Small-angle neutron scattering data were collected at the ISIS neutron and muon source (Oxford, U.K.) on the ZOOM beam-line, with an observed Q-range of 2 × 10^–3^ Å^–1^ < *Q* < 0.45 Å^–1^. Two-dimensional (2D) data were radially averaged, and standard reduction procedures (subtraction of empty cell and solvent contribution) were applied. The fitting procedure of the obtained scattering curves was performed with the NIST package with IGOR Pro (WaveMetrics, Inc.).^27^

### 2.6 Differential Scanning Calorimetry (DSC)

DSC measurements were performed using a TA Q2000 (New Castle) apparatus. Steel pans were used as sample holders containing 15–20 mg of sample. The analysis was performed in heating mode between −80 and +25 °C, using a 0.5 °C/min heating ramp. For the calculation of the free water content (FWC) of each sample, the following formula was used
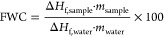
1where Δ*H*_f,sample_ and Δ*H*_f,water_ are the enthalpy of fusion (J/g) of water in the sample and of pure water, respectively. *m*_sample_ and *m*_water_ are the weight of the sample and the nominal amount of water in the sample, respectively (in g). Δ*H*_f,sample_ was obtained by integrating the bands in the −20 to +5 °C temperature range. Δ*H*_f,water_ was obtained from the literature.^28^

### 2.7 Rheometry

Rheological measurements were carried out on a TA DHR3 rheometer, which operated in controlled shear stress mode, using a plate–plate geometry (Flat Plate 60 mm diameter) and a Peltier system for temperature control. All of the measurements were carried out at 25 °C. The gap between the plates at zero radial position was always maintained at 500 μm. The cell was closed by lowering the head to the measuring position in the *z*-axis force-controlled mode; flow curves were collected by measuring the viscosity values under the application of a shear rate logarithmic ramp in the range 1–10^3^ s^–1^.

### 2.8 Optical Microscopy

Optical images were collected with an inverted optical microscope (Diaphot 300, Nikon) equipped with a digital camera (Nikon Digital Sight DS-U1). The objective magnification used was 20×. The image analysis was performed using the ACT 2U software by Nikon.

### 2.9 Confocal Laser Scanning Microscopy (CLSM)

CLSM imaging was performed with a Leica TCS SP8 confocal microscope (Leica Microsystems GmbH, Wetzlar, Germany). Wells were used as sample holders (Lab-Tek Chambered 1.0 Borosilicate Coverglass System, Nalge Nunc International, Rochester, NY). A water-immersion 63× objective was used to image the samples. Rhodamine-B was used as a probe, and it was excited at 561 nm with a DPSS laser. A hybrid SMD detector was used for the fluorescence emission in the 571–600 nm range.

### 2.10 Confocal Raman Microscopy

Raman analyses and mapping were performed on a Renishaw InvIa Qontor confocal MicroRaman system equipped with 785 nm (solid-state type, IPS R-type NIR785, 100 mW, 1200 L/mm grating) and 532 nm (Nd:YAG solid-state type, 50 mW, 1800 L/mm grating) lasers, front-illuminated CCD camera (256 × 1024 px, working temp. −70 °C), and a research-grade Leica DM 2700 microscope equipped with LWD 50× (NA 0.55, WD 8.0 mm), LWD 100× (NA 0.75, WD 4.7 mm), and 100× (NA 0.85, WD 0.27 mm) objectives.

Samples were prepared by placing a small amount of product between two microscopy glass slides. References for pure compounds were collected using the 785 nm excitation wavelength for Soluplus and 532 nm excitation wavelength for the PRMs. Raman spectra were recorded in the 300–3700 cm^–1^ wavenumber range using the extended range mode. Acquisition times for pure compounds varied between 10 and 40 s. 2D maps were acquired in static range measurement and high confocality mode using the LWD 50× objective and the 532 nm laser. 2D maps were acquired in steps of 1 μm along the *x*–*y-*axis with 1 s acquisition time per point. Raw data were processed using the Renishaw software WiRE v.5.2 for baseline correction, peak fitting, and map generation.

## 3 Results and Discussion

### 3.1 NMR Characterization of Soluplus Macromolecule

**Figure 1:**
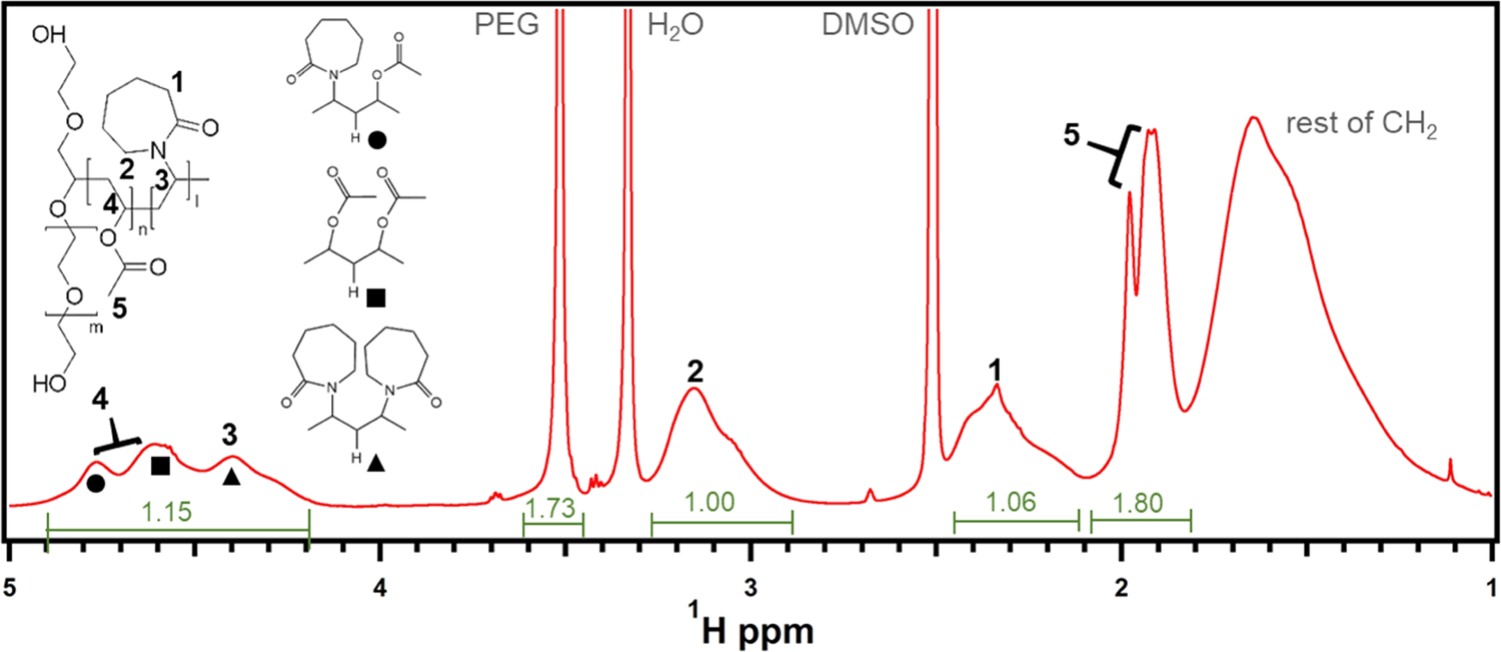
Soluplus 30 mg/mL in DMSO-*d*_6_,^1^H NMR spectrum.

The molecular structure of Soluplus, its monomeric components, and their relative ratio in the macromolecule were elucidated by means of NMR experiments. The comparison between ^1^H NMR spectra (Figure 1) and {^1^H–^13^C}-HSQC, {^1^H–^1^H}-NOESY, and {^1^H–^1^H}-COSY correlation maps (Figures S1–S3) allowed for a clear band assignment and provided information about the assembly of VAc and VCL units on the graft. The ^1^H NMR peak assignment is presented in Figure 1 and is completely consistent with the expected Soluplus structure as described by the manufacturer. The {^1^H–^13^C}-HSQC map helped in the assignment of the CH_3_ moieties of the copolymer (showed as opposite phased, colored in blue in the map of Figure S2) and the CH and CH_2_ moieties (colored in red). In addition, the actual ethylene oxide/vinyl acetate/vinyl caprolactam (EO/VAc/VCL) ratio was calculated by opportunely integrating ^1^H NMR peaks. The resulting weight percentage of the three components is 13% EO, 34% VAc, and 53% VCL, in good agreement with the one reported by the supplier (13% EO, 30% VAc, and 57% VCL).^1^ The determination of the number of grafting sites per chain, as previously reported for a similar polymer, i.e., PEG-*g*-PVAc,^14^ was attempted through inverse-gated proton-decoupled ^13^C NMR. Unfortunately, in this case, it was not possible to find a detectable band clearly allied with the resonance of the PEG’s grafted methynes. Such result is however compatible with a very low degree of grafting, where the PEG backbone is grafted with a very small number of PVAc-co-PVCL chains, being it below the intrinsic detection limit of the technique. {^1^H–^1^H}-NOESY and {^1^H–^1^H}-COSY maps were used to gain insights into the block or random nature of the P(VAc-co-VCL) portion. The presence of strong correlation signals (areas indicated with black frames in Figures S3 and S4) between almost all vinyl caprolactam and vinyl acetate resonances suggested a prevalently random or alternate distribution, excluding a block configuration. A more quantitative evaluation of the degree of blockiness of the P(VAc-*co*-VCL) moiety was thus obtained by following an approach similar to the one reported by Moritani et al.^29^ for partly hydrolyzed poly(vinyl alcohol). A blockiness index (*η*_B_) for binary copolymers can be defined as the ratio between the fraction of alternate dyads (e.g., VAc-VCL) and the run fraction of the copolymer as purely random. It can take values between 0 for block copolymers and 2 for perfectly alternate ones, while a value of *η*_B_ = 1 is indicative of a completely random distribution. In our case, the fraction of VAc monomers in alternate (VAc, VCL) dyads was estimated through the deconvolution of the ^1^H NMR band in the 4–5 ppm range in four Gaussian curves—one per each possible dyad—and their relative assignment and integration. A blockiness index *η*_B_ ≈ 0.8 was obtained, suggesting a prevalently random configuration.

### 3.2 Self-Assembly Properties and Nanostructure of Soluplus in Water

**Figure 2:**
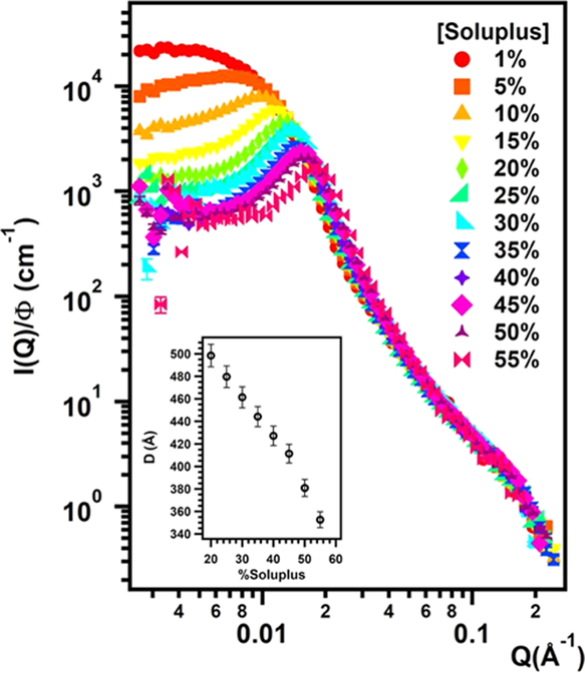
SANS curves normalized by the volume fraction for samples containing Soluplus in D_2_O with concentrations from 1 to 55% in steps of 5%. Inset: intermicellar distance, *D* (obtained from the correlation peak position) vs polymer concentration.

Though Soluplus applicability as a drug carrier has been quite extensively studied, the physicochemical properties and structural characterization of its self-assembly properties have just been preliminarily reported by Mateos et al.^30^ for a limited range of Soluplus/water binary system concentrations (1–20% w/w). Here, we report a comprehensive study of the self-assembling properties, employed to analyze SANS data collected on a wide concentration range (1–55% w/w) of Soluplus/D_2_O binary systems. Prior to the physicochemical investigation of the self-assembling properties of Soluplus, we investigated its CMC surface tension, see Figure S4. The CMC value of 7.6 ± 0.1 mg/L is in very good agreement with the value reported from the manufacturer and literature.^1,31^Figure 2 shows SANS scattering patterns for samples with concentrations ranging from 1 to 55% w/w, normalized by the theoretical volume fraction of the dispersed phase after subtracting the incoherent background scattering intensity from the reduced data. Non-normalized SANS patterns are reported in Figure S5. A first qualitative analysis of the experimental curves reveals a downturn of the scattering intensity *I*(*Q*) in the low-*Q* region for concentrations higher than 1% and the further appearance of an interaction peak that grows in intensity and moves to higher *Q* values as the polymer content increases. The shift of the interaction peak toward higher *Q* values is evidence of the decrease in the interparticle distance, as particles come into closer contact. For all curves, the intensity decrease in the Porod region (high *Q* values in a log *I*/log *Q* plot) follows a *Q*^–2^ power law, typical of polymer coils.

Our quantitative analysis started from the 1% Soluplus, i.e., the most dilute sample. A Kratky plot for this curve, *Q*^2^ × *I*(*Q*) vs *Q* (see Figure S6), shows that the sample’s scattering objects are globular.^32^ The radius of gyration, *R*_G_, was then calculated from the SANS data through a Guinier plot, ln(*I*(*Q*)) vs *Q*^2^ (see Figure S7), using the well-known approximation^32^
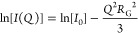
2which led to *R*_G_ = 177 ± 5 Å. Thanks to the relationship *R*_G_^2^ = 3/5*R*^2^ for spherical objects, the radius of the micelle^33^*R* = 228.5 Å consistent with *R*_H_ values reported in the literature was obtained.^23,25^ From the interaction peak position, a real distance among scattering “objects” can be obtained from the relation *D* = 2π/*Q* and *D* values that are reported against Soluplus concentration in the inset of Figure 2.

**Figure 3:**
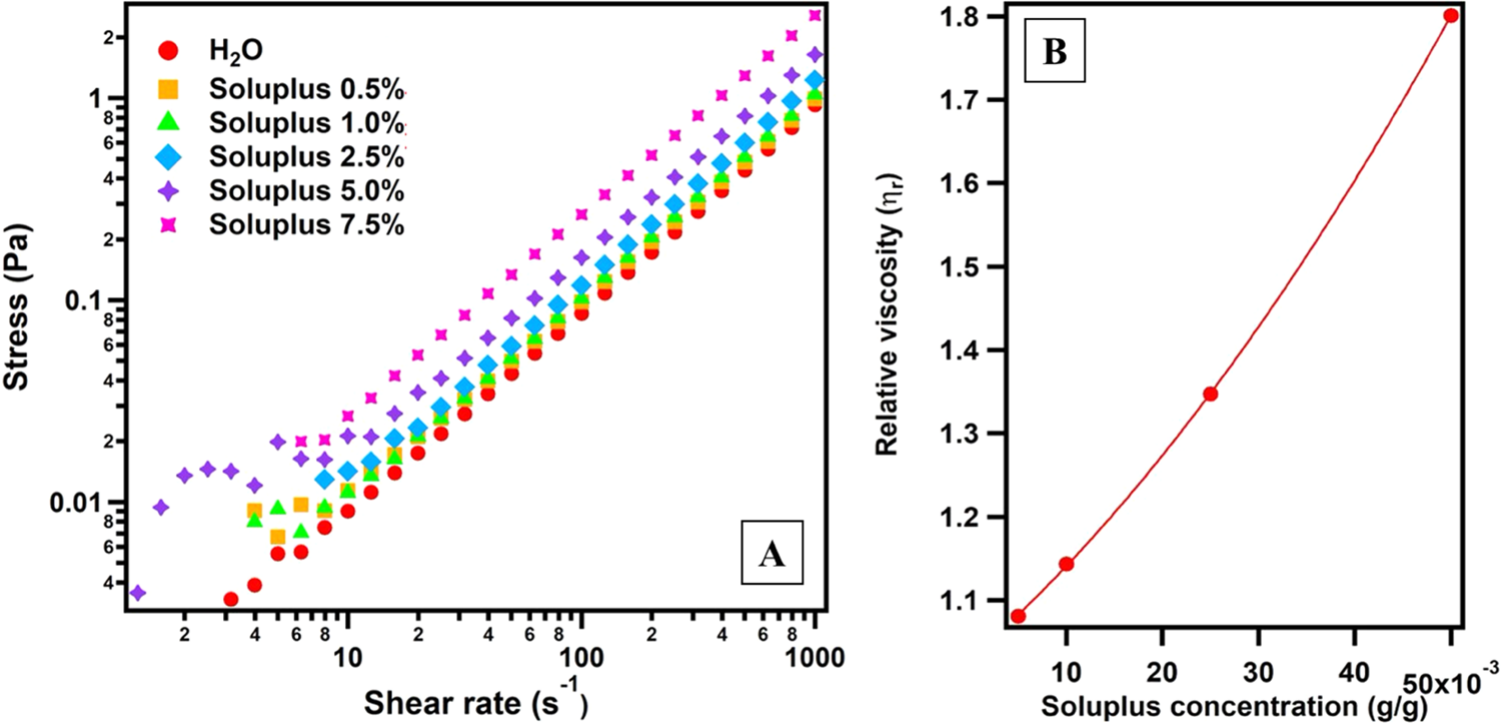
(A) Flow curves of Soluplus in water for samples from *C* = 0.5–7.5% w/w. (B) Relative viscosity of Soluplus 1, 2.5, 5, and 7.5% w/w samples as obtained from the flow curves vs Soluplus concentration, fitted to eq 3.

For soft, highly hydrated colloidal particles like Soluplus micelles, the volume fraction usually cannot be calculated directly from the sample composition; rather, the effective volume fraction (Φ_H_) needs to be considered. A common method to evaluate Φ_H_ for such systems is through rheometry, by measuring the relative viscosity, η_r_. According to the Batchelor–Einstein equation, in dilute conditions, Φ_H_ is related to the relative viscosity of the colloidal suspension according to the equation^34−37^
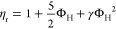
3We define Φ_H_ = *kC*, where *C* is the weight fraction of the sample and *k* is a constant of proportionality. *k* and γ can be extracted by plotting η_r_ with weight concentration and then fitting with Batchelor–Einstein equation, substituting Φ_H_ with *kC*. Besides the determination of k that leads to Φ_H_, γ coefficient can give information on the interparticle interactions. For Brownian hard spheres, γ is expected to be comprised between 5.9 and 6.2, while higher values are indicative of attraction between the colloidal particles. The flow curves of Soluplus in water from 1 to 7.5% w/w are presented in Figure 3A. It is evident that the samples up to 7.5% w/w exhibit Newtonian fluid behavior and their viscosity remains constant over the accessible shear rate window. The relative viscosity of the dilute solutions was plotted against polymer concentration (Figure 3B). Best fitting with eq 3 returns 1 + (10 ± 0.5)*C* + (107.1 ± 0.7)*C*^2^ leading to *k* = 4.0 ± 0.2 and γ = 6.7 ± 0.4. The analysis is thus suggesting that Φ_H_ is about 4 times the theoretical value obtained by the weight fraction of the suspensions (see Table S1). The value of γ coefficient is higher than the one expected for hard spheres, suggesting the presence of additional attractive interactions between Soluplus micelles. The obtained value of Φ_H_ was used for the fitting of SANS patterns of the diluted Soluplus water solutions (1 and 5% w/w).

**Figure 4:**
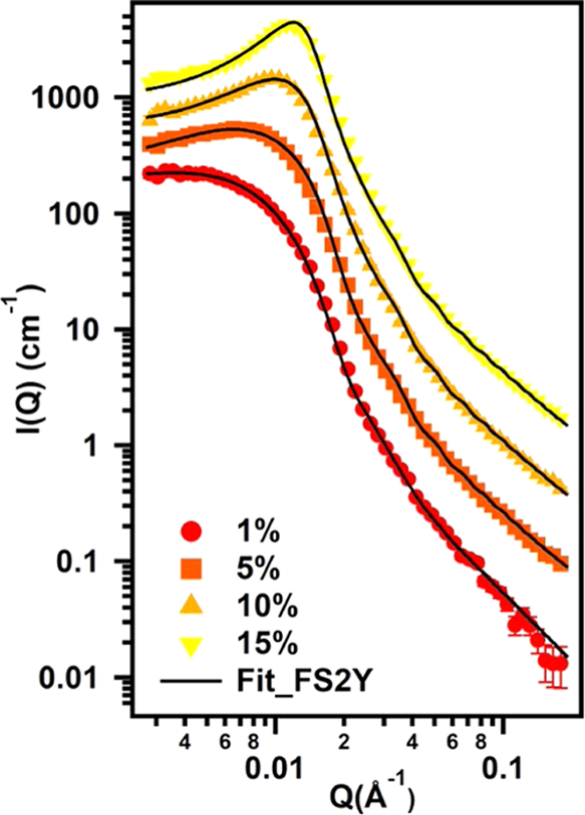
SANS patterns obtained for the samples containing 1–15% Soluplus in D_2_O. Markers represent experimental points and solid line represents fitting with the fuzzy sphere—2Y model (eq 4) as explained in the main text. Curves were offset along the *y*-axis for presentation purposes.

The fitting procedure for Soluplus 1–5% SANS patterns led to the results summarized in Figure 4 and Table 1. SANS patterns were modeled according to a fuzzy sphere (FS) form factor with a double Yukawa (2Y) interaction potential,^38,39^ and an additional Lorentzian term was added to this model. The total scattering intensity is given by eq 4
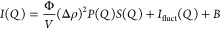
4where Δ_ρ_ is the scattering length density (SLD) difference between the sphere and the solvent, Φ is the volume fraction of particles, *V* is the sphere volume, *P*(*Q*) is the fuzzy sphere form factor, *S*(*Q*) is the 2Y structure factor, *I*_fluct_ accounts for the Lorentzian function, and *B* is the contribution of the incoherent background. All of the mathematical functions are given in the Supporting Information (SI) (eqs S2–S7) along with a detailed explanation of the fitting parameters. Briefly, this model describes micelles as spherical particles in which the polymer’s density is gradually decaying from the particle’s center to its surface. The micelle is thus composed of a more compact core and a corona with a fuzzy interface, which in the present case should be constituted, respectively, by the slightly more hydrophobic P(VAc-*co*-VCL) grafts and the more hydrophilic PEG backbone portions of the polymer macromolecule. The Lorentzian term is added to the model to describe the ensemble average correlations in the polymer network.^38^ For Soluplus 1 and 5%, Φ_H_ calculated from rheology was used as the volume fraction for the fitting procedure. For 10 and 15%, the volume fraction was left as a free parameter. In those two samples, the concentration was higher than the concentration range where the Bachelor–Einstein method was employed. Thus, using the approach adopted for more diluted samples to evaluate Φ_H_ was considered unfeasible for samples having a concentration higher than 7.5%. Moreover, it would have led to extremely high-volume fractions, which are unphysical. Micellar size can be obtained from fitting parameters as *R* = *R*_core_ + 2σ.^40^ The fitting shows that the micellar radius, *R*, is around 22.4 nm and almost constant in the 1–15% polymer concentration range, in agreement with the results by Mateos et al.^30^ The correlation length, ξ, of the Lorentzian term is reduced from 6 nm for 1% to 4.4, 3.8, and 3.1 nm for 5, 10, and 15%, respectively. This was expected and it is in agreement with literature data on PNiPAM microgels, since, as the effective volume fraction increases, polymer chain fluctuations are restricted to a smaller length scale.^38^ Regarding the structure factor parameters, the attraction and repulsion ranges (inversely proportional to the range parameters reported in Table 1) decrease with increasing polymer concentration, as expected, since the micelles are coming into closer contact. The 2Y interaction potential with a long-range repulsion and a short-range attraction component is consistent with attractive interactions that are also present between Soluplus micelles (γ coefficient extracted from rheology analysis), arising from the interchain attraction of polymer chains in the micellar corona.

**Table 1:** Structural Parameters Obtained by Fitting the 1–15% of Soluplus/D_2_O SANS Curves with the Fuzzy Sphere 2-Yukawa (FS2Y) Model (Equation 4)^a^

Soluplus (% w/w)	1	5	10	15
volume fraction	0.04	0.2	0.25	0.28
core radius, *R*_core_ (Å)	173 ± 6	178 ± 7	173 ± 7	171 ± 3
core polydispersity	0.27	0.27	0.34	0.38
fuzziness, σ (Å)	25	25	23	23
core SLD (Å^–2^)	4.95 × 10^–6^	4.8 × 10^–6^	4.4 × 10^–6^	4.2 × 10^–6^
solvent SLD (Å^–2^)	6.4 × 10^–6^	6.4 × 10^–6^	6.4 × 10^–6^	6.4 × 10^–6^
Lorentzian scale	2	5	7.2	7.2
Lorentzian length (Å)	60	44	38	31
attraction strength	3.0 ± 0.3	4.06 ± 0.05	4.0 ± 0.1	4.5 ± 0.1
attraction range parameter	19 ± 2	28.0 ± 0.1	29.0 ± 0.3	30.0 ± 0.1
repulsion strength	–0.40 ± 0.01	–0.70 ± 0.01	–2.8 ± 0.1	–6.7 ± 0.1
repulsion range parameter	1.19 ± 0.01	1.20 ± 0.01	2.2 ± 0.1	2.3 ± 0.1

a The parameters for which no error is given were kept fixed during the fitting procedure.

The inset of Figure 2 shows that for a Soluplus concentration of 35%, the correlation peak is centered at *Q* = 0.014 Å^–1^, which corresponds to a real-space center-to-center average distance (*D* = 2π/*Q* = 44 nm) twice the radius R obtained from the fitting. This shows that micelles are statistically in contact with each other at this concentration. Consequently, at high Soluplus concentrations (≥20%), the fuzzy sphere model was unsuitable to fit the experimental curves.

**Figure 5:**
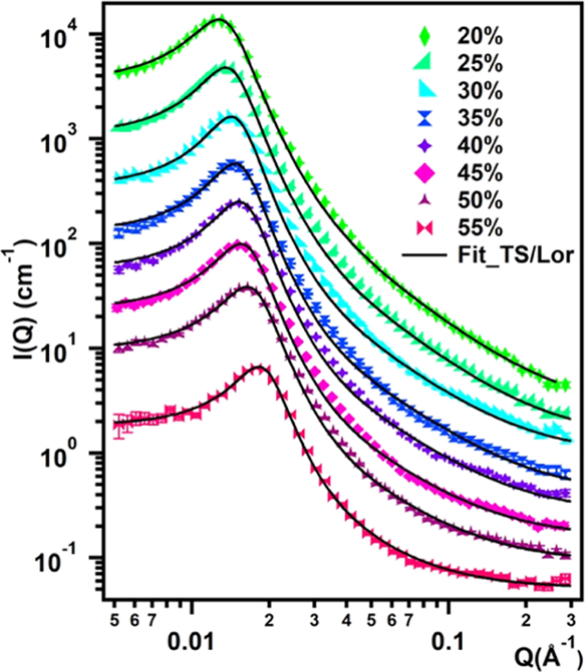
SANS curves obtained for samples containing 20–55% Soluplus in D_2_O. Markers represent experimental points and solid lines represents best fitting curves obtained with the TS/Lor model (eq 5). Curves were offset along the *y*-axis for presentation purposes.

Several fitting models were used to describe the Soluplus/D_2_O scattering patterns with concentration ≥20%, including polydisperse spheres or fuzzy spheres as the form factor, and 2Y or hard-sphere interaction potentials. However, the best and most reliable fitting was obtained using the Teubner–Strey (TS) model plus an additional Lorentzian term (TS/Lor model). The fitting curves are reported in Figure 5 together with the experimental data. The model’s mathematical function is given by eq 5
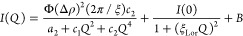
5where Φ is the volume fraction of particles; Δ_ρ_ is the scattering length density (SLD) difference between the spherical particle and the solvent; parameters α_2_, *c*_1_, and *c*_2_ are coefficients that can be defined in terms of two characteristic lengths, a correlation length (ξ), and periodicity (*d*); *I*(0) is the intensity at *Q* = 0; ξ_Lor_ is the correlation length of the Lorentzian term; and *B* is the incoherent background. A more detailed explanation of the fitting parameters can be found in the SI (eqs S9–S15). The TS model has been originally developed by Teubner and Strey to describe three- (or more) component microemulsion systems.^41^ Since then, it has been successfully extended to interpret the scattering behavior of micellar systems,^42,43^ and two-component systems,^44−46^ in the case where the microscopic structure of the system contains amphiphile-rich and water-rich discrete domains. For Soluplus/D_2_O binary mixtures, as the concentration of the amphiphilic polymer increases, micelles start to interpenetrate into each other, yielding something that closely resembles a bicontinuous structure with water-rich and amphiphile-rich domains. Additionally, a Lorentzian term was included in the fitting procedure (similarly to the fuzzy sphere—2Y model for diluted samples) that accounts for the enhanced density fluctuations of the polymer chains at the micellar surface. The obtained fitting parameters and the calculated amphiphilicity factor (*f*_α_) can be seen in Table 2. The ξ and *d* parameters generally decreased, as expected for micelles coming into closer contact. The amphiphilicity factor, *f*_α_, that can be derived from ξ and *d* using eq S15(47), describes the amphiphile’s “strength” or “quality” and the degree of order in the system. The amphiphilicity factor can vary between −1 < *f*_α_ < 1, where *f*_α_ = −1 corresponds to an ordered system and *f*_α_ = 1 corresponds to a disordered phase. When *f*_α_ is negative and approaches −1, the scattering intensity exhibits well-defined peaks at *I*(*q*) ≠ 0 and the system can be characterized as a “strong” amphiphile with ordered domains, as it happens for lamellar phases. Interestingly, for Soluplus/D_2_O mixtures, the calculated amphiphilicity factor, *f*_α_, was constant and its value varied between −0.87 and −0.90, close to *f*_α_ = −1, that is characteristic of strong amphiphiles and ordered systems. For micellar systems, as the present Soluplus/D_2_O ones, the periodicity parameter (*d*) obtained from the TS model represents the intermicellar distance.^45^*d* Values are in relatively good agreement with the intermicellar distance (*D*) values evaluated through the relation 2π/*Q*_max_ (see Table 2). The fact that we obtained values that perfectly agree with the trend of those obtained at lower Soluplus concentrations is an indication of the reliability of the TS model in the present context.

**Table 2:** Structural Parameters Obtained by Fitting the 20–55% Soluplus/D_2_O SANS Curves with the TS/Lor Model (Equation 5)^a^

Soluplus (% w/w)	20	25	30	35	40	45	50	55
ξ (Å)	286 ± 2	293 ± 3	292 ± 4	276 ± 4	264 ± 4	250 ± 4	235 ± 3	223 ± 3
*d* (Å)	475 ± 1	449 ± 1	428 ± 1	414 ± 1	399 ± 1	386 ± 1	370 ± 1	334 ± 1
*f*_α_	–0.87	–0.89	–0.90	–0.89	–0.89	–0.89	–0.88	–0.89
Lorentzian scale	7	7	4.5	5.5	4.5	4.5	4.5	2
Lorentzian length (Å)	30	29	29	25	26	27	28	43
*D* (Å)	498.41	479.76	461.43	444.13	427.21	411.26	380.84	352.61

a The parameters for which no error is given were kept fixed during the fitting procedure. The amphiphilicity factor, *f*_α_, was calculated with eq S15 and the intermicellar distance, *D*, was calculated from 2π/*Q*_max_ using the position of the correlation peak of the scattering curves.

More information on the micellar phases’ ordering at high concentration can be accessed by further exploiting the position of the correlation peaks present in the SANS patterns. The intermicellar distance, *D* (or *d*), depends on the amphiphile concentration and, for globular micelles following a face-centered-cubic ordering, the mean distance between micelles is given by eq 6(48)
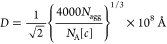
6where *c* is the molar concentration of the polymer, *N*_agg_ is the aggregation number, and *N*_A_ is Avogadro’s number. Figure S8 reports the intermicellar distance, *D* or *d* (obtained after fitting with the TS model), versus the reverse cubic root of the polymer concentration and the points can be fitted to a straight line. The fact that Soluplus in the 20–45% concentration range is linearly proportional to the reverse cubic root of the polymer concentration is an indication of a face-centered-cubic ordering of Soluplus micelles, supporting the finding of *f*_α_ close to the value of ordered systems and concentration-independent globular shape across the concentration range studied.^49^ The aggregation number, *N*_agg_, of the micellar system can be then calculated from the slope of the linear fitting in Figure S8, leading to *N*_agg_ = 32 ± 1.

**Figure 6:**
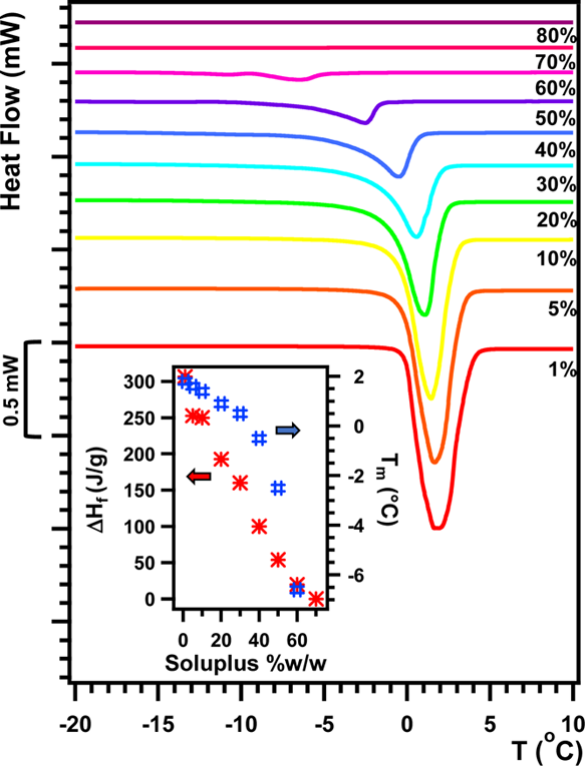
DSC thermograms for Soluplus samples in H_2_O. Inset: enthalpies of fusion (Δ*H*_f_, J/g) and melting temperatures (*T*_m_, °C) as a function of polymer concentration.

*N*_agg_ value was further confirmed by thermal analyses. DSC thermograms for the 1–80% Soluplus in H_2_O are shown in Figure 6 (for ease of comparison with the SANS results, the same set of experiments was also performed in D_2_O solutions; see Figure S9). Integrating the endothermic peak due to the melting transition of water (around 0 °C) yields the enthalpy of fusion, Δ*H*_f_, of each sample, and the free water content (FWC) by means of eq 1. For the sample containing 1% Soluplus, FWC = 93% (for both H_2_O and D_2_O) of the total water content in the sample (calculations are reported in the SI); therefore, the remaining 7% water is represented by nonfreezable water, bound to the hydrophilic moieties of the polymer. *N*_agg_ can be approximately obtained by dividing the total volume of a micelle (*V*_micelle_) by that of each polymer chain (*V*_chain_), according to eq S18. Following this approach, we obtain *N*_agg_ = 30 ± 1, in perfect agreement with the value obtained from SANS.

Furthermore, the analysis of DSC data offers information on the hydration of polymer chains. The inset in Figure 6 shows the dependence of Δ*H*_f_ on polymer concentration. As expected, increasing polymer concentration up to 70%, Δ*H*_f_ (and, as a consequence, FWC) decreases, until there is no more free water in the sample (in both H_2_O and D_2_O, see Figure S9). If we initially consider that only PEG is hydrated, this gives seven to eight water molecules per EO unit (see SI for the calculations). This number is significantly higher than the number reported in literature (three to four H_2_O per EO unit),^50^ indicating that likely also VCL units of the graft chain could be hydrated. This finding is not surprising, since VCL is mostly hydrophilic and as hydrated as PEG units.^51^ The high average hydration of Soluplus chains can thus account for the large values obtained for the core SLD in the SANS experiments, meaning that water can penetrate deep into the micelles, as it happens for PNiPAM microgels, which share several structural features with Soluplus supramolecular aggregates,^52−54^ and as reported for other amphiphilic PVCL-based microgels, where increasing the amount of the hydrophilic PVCL segment in the copolymer increased the swelling of the system.^55^

### 3.3 Soluplus as an Encapsulating Agent for Fragrances in Aqueous Media

**Figure 7:**
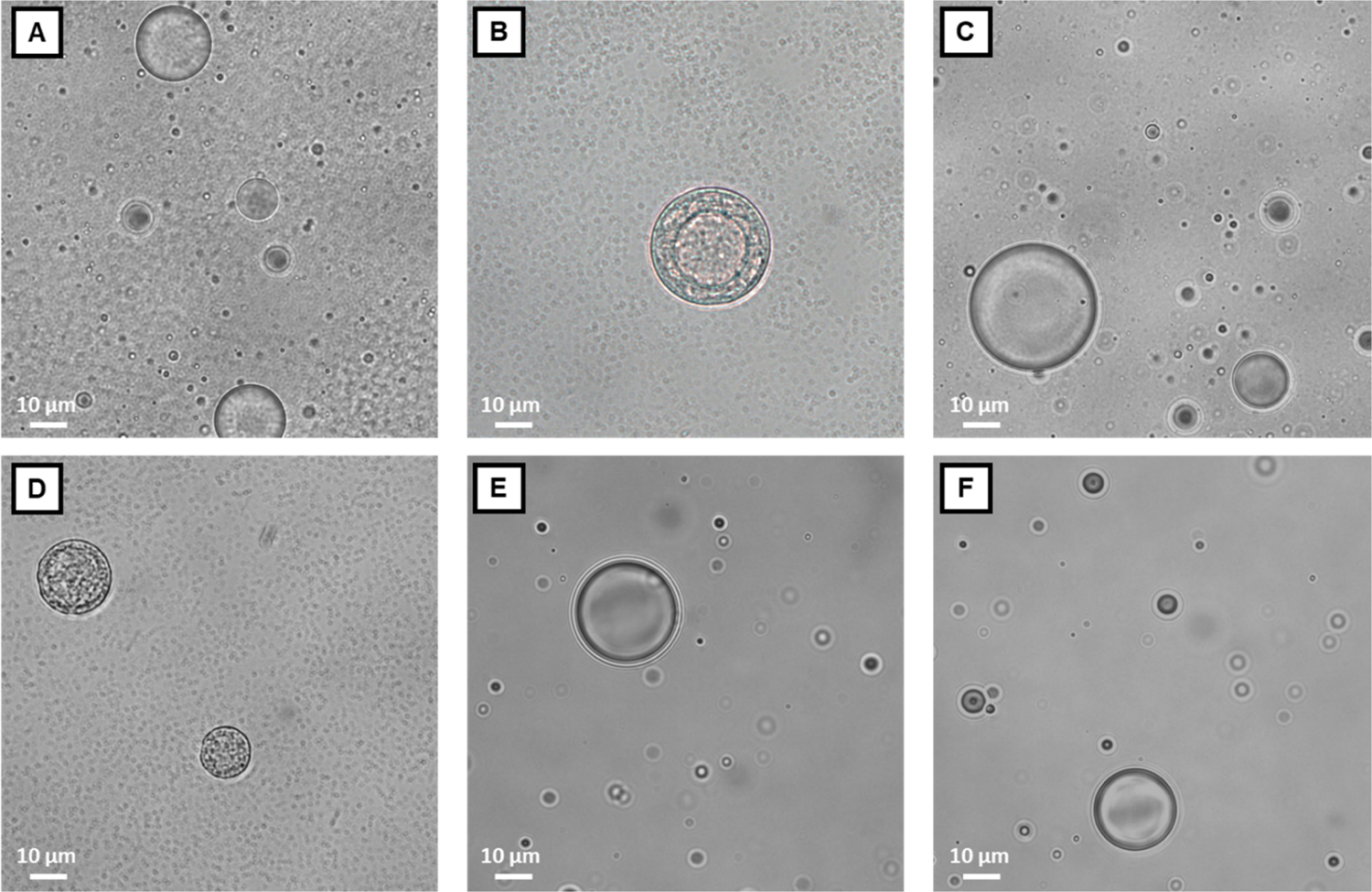
Optical microscope images of 94% w/w water, 5% polymer, and 1% of each of the perfumes: (A) l-carvone, (B) linalool, (C) florhydral, (D) β-citronellol, (E) α-pinene, and (F) *R*-limonene.

To assess the capability of Soluplus as an encapsulating agent for fragrances in aqueous media, a series of seven PRMs having not only different hydrophobicities but also different molecular characteristics (i.e., functional groups, molecular conformation, bulkiness) was selected. As said, hydrophobicity of molecules can be expressed by log *K*_ow_, a parameter commonly used to classify fragrances in several perfume encapsulation studies.^56−60^ The selected PRMs were 2-phenyl ethanol (PE, log *K*_ow_ = 1.36), l-carvone (CAR, log *K*_ow_ = 2.74), linalool (LIN, log *K*_ow_ = 2.97), florhydral (FLO, log *K*_ow_ = 3.02), β-citronellol (CIT, log *K*_ow_ = 3.3), α-pinene (PIN, log *K*_ow_ = 4.44), and *R*-limonene (LIM, log *K*_ow_ = 4.57) (molecular structures in Scheme 1). Since several types of industrial formulations are composed of around 90% w/w water and only few percent of the encapsulating agent and the active compounds, the tests were carried out with Soluplus solutions containing 94% water, 5% polymer, and 1% perfume. After the fragrance addition, except for PE, all samples looked milky, suggesting the presence of dispersed objects bigger than 300−500 nm. Indeed, optical microscopy indicated the presence of micron-sized spherical particles, as shown in the micrographs in Figure 7, which report six out of the seven PRM-based systems investigated. Such systems are usually metastable but can be included in an industrial formulation taking advantage of structures that increase the viscosity. In our studies, PRM/Soluplus/water systems were stable for more than 3 months, and no coalescence of the supramolecular structures was observed during the whole time in which samples were investigated through optical and confocal microscopy.

**Figure 8:**
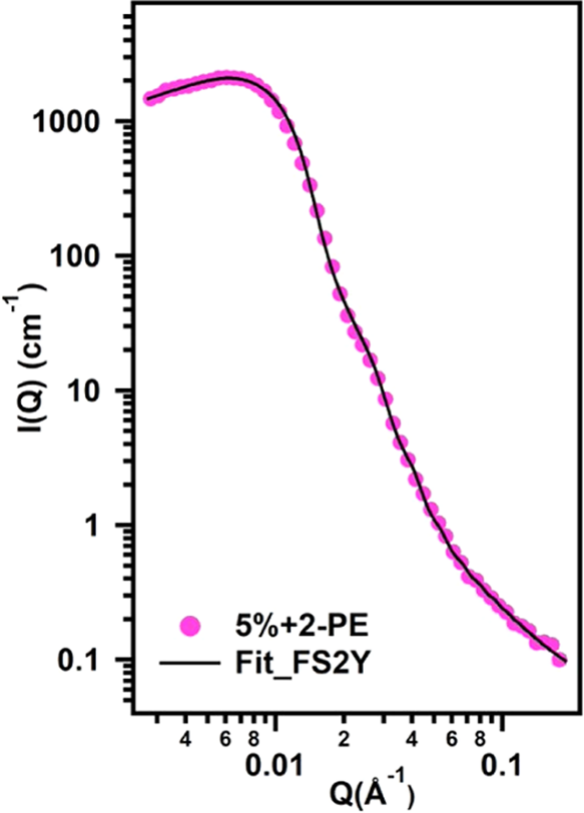
SANS patterns obtained for the sample containing 5% Soluplus/1% PE in D_2_O. Markers represent experimental points, and solid line represents fitting with the FS2Y model as explained in the main text.

In fact, the sample containing 5% Soluplus and 1% PE was a macroscopically homogeneous and transparent single phased, evidencing the absence of micron-sized objects. Therefore, this sample was further studied by means of SANS (Figure 8). The scattering pattern was fitted with the same fuzzy sphere 2-Yukawa (FS2Y) model employed for Soluplus micelles without PRMs (eq 4). The obtained fitting parameters can be found in Table S4. The micellar core radius increased from 17.8 (± 0.7) to 22.2 nm (± 0.4) upon the addition of PE to 5% Soluplus. This result, together with the decrease of the core SLD from 4.8 to 4.0 (× 10^–6^) Å^–2^ (the SLDs of pure components are reported in Table S2), indicated that the PRM was actually solubilized inside the polymeric micelle, likely replacing some D_2_O molecules. Low log *K*_ow_ fragrances like PE tend to partition themselves between the dispersed, more hydrophobic phase and the aqueous bulk solvent, thus causing a slight swelling of micelles.^61^ Overall, the behavior of PE, when added to Soluplus micelles in water is consistent with the Soluplus micelles core being not purely hydrophobic, as evidenced by the SANS characterization. In fact, the abundant presence of VCL units and the significant amount of penetrating water molecules create an environment preferably suited to encapsulate/solubilize low hydrophobicity PRMs, such as PE. The {^1^H–^1^H}-NOESY correlation map of the same sample (5% Soluplus/1% PE in D_2_O) (Figure S10) gives more insights into the interaction of the fragrance with the polymer. Apparently, no strong correlation between the protons of PE and those of Soluplus is present. This can be explained considering that PE molecules were included in the micelle in the form of tiny droplets or nanodomains. The perfume is not molecularly distributed in the micelles but is instead forming a core (single droplet) or more pools of the solvent distributed in the volume of the micelles. From the NOESY experiment, it is evident that 2-phenyl ethanol molecules mostly interact with themselves, with only a few of them being spatially in very close contact with the polymer, thus causing a slight swelling of the micelle.

Samples prepared with the remaining PRMs were investigated with CLSM (Figure 9), using rhodamine-labeled Soluplus, and confocal Raman microscopy (CRM). Reference Raman spectra of pure Soluplus and spectra of pure PRMs are reported in Figures S11–S17. 2D Raman mapping was performed to localize the perfume and water in the aggregates (Figures 10 and S18).

**Figure 9:**
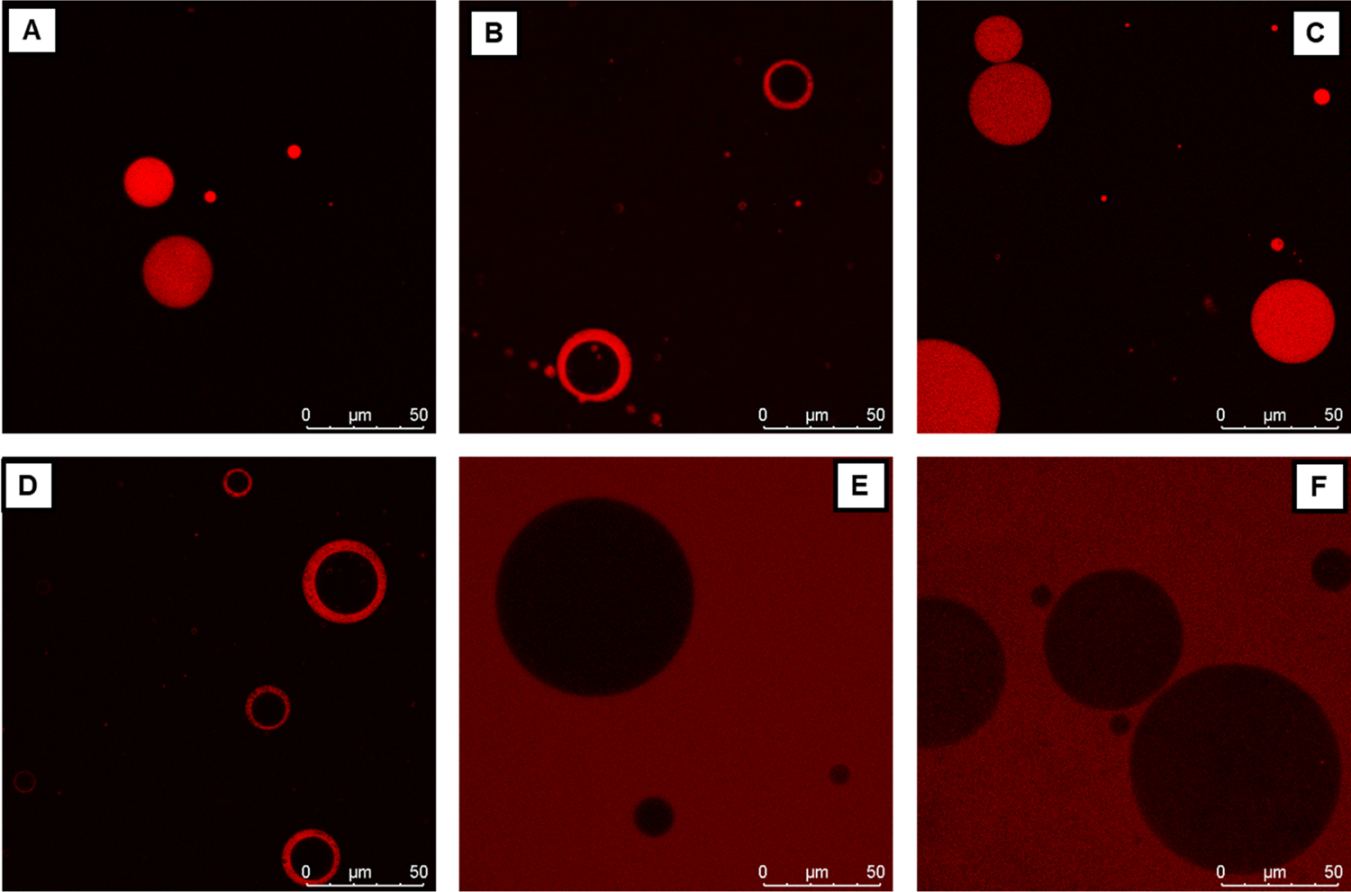
Confocal scanning laser microscopy images (63× water-immersion objective) of 94% w/w water, 5% polymer, and 1% of each of the perfumes: (A) CAR, (B) LIN, (C) FLO, (D) CIT, (E) PIN, and (F) LIM.

**Figure 10:**
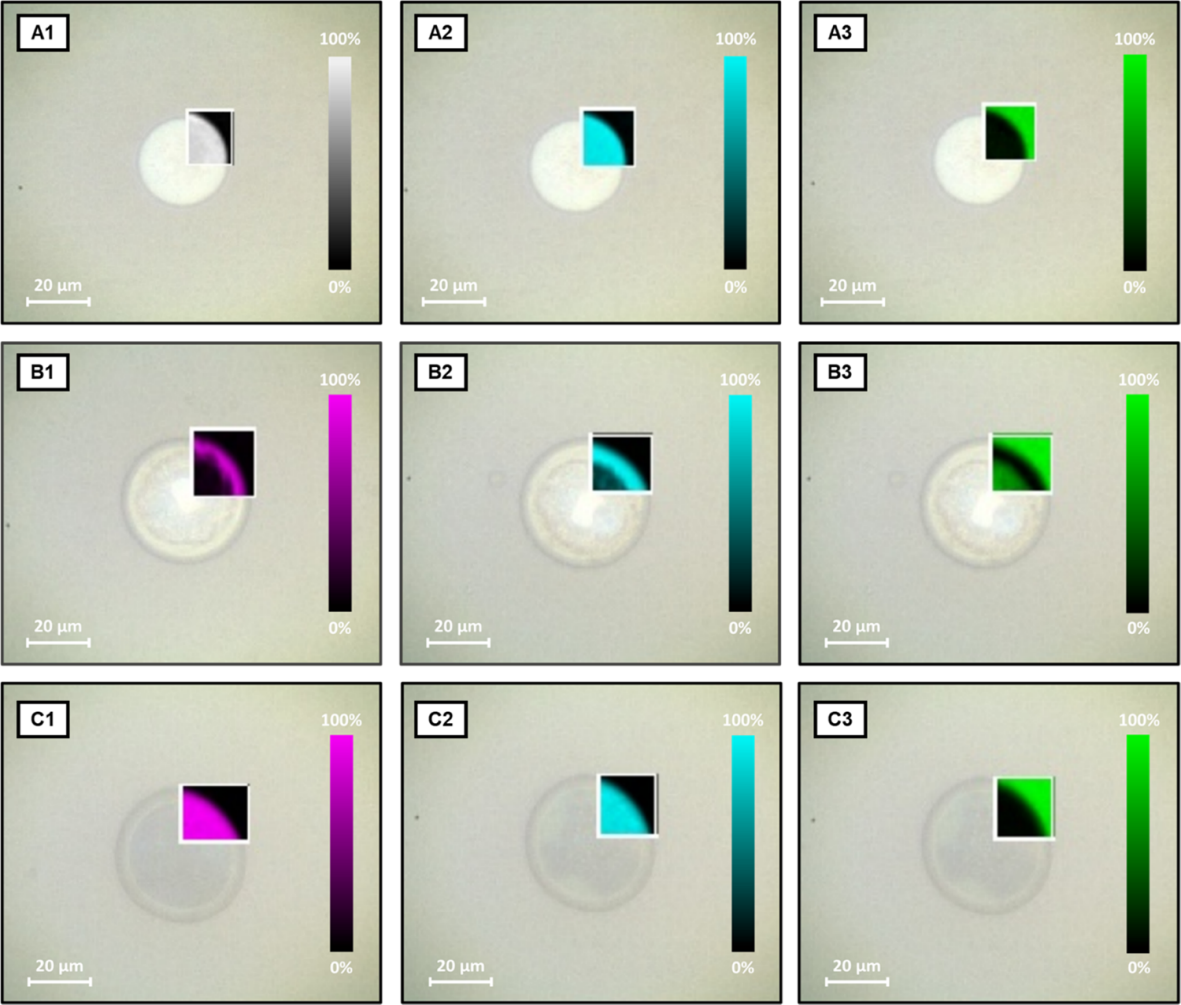
Raman 2D mapping (LWD 50× objective) of 94% w/w water, 5% polymer, and 1% of each of the perfumes: (A) FLO, (B) LIN, and (C) PIN. The different colors represent tracking of the different Raman signals. White signal: tracking at 1000 cm^–1^ (aromatic ring stretching). Pink signal: tracking at 1640 cm^–1^ (C = C stretching band). Blue signal: Tracking at 2920 cm^–1^ (C–H stretching band). Green signal: Tracking at 3400 cm^–1^ (O–H stretching band). 2D Raman maps with CAR, CIT, and LIM report similar observations to the ones made for FLO, LIN, and PIN, respectively, and can be found in Figure S18 in the Supporting Information.

For FLO, CAR, LIN, and CIT, CLSM images revealed the presence of large polymeric aggregates of size between 10 and 100 μm. FLO and CAR led to the formation of matrix-type polymeric capsules, where the polymer is embedded in the whole capsule area. FLO was, then, tracked with CRM in the capsules, using the Raman signal of its aromatic ring vibrations at around 1000 cm^–1^, and the 2D mapping generated can be seen in Figure 10A1. This evidenced that both FLO and CAR are present in the whole volume of polymeric matrix-type capsules together with Soluplus.

The case of LIN and CIT stands out due to the presence of objects remindful of polymersomes or multiple w/o/w emulsions, clearly visible in CLSM images (Figure 9B,D). A further insight into these microstructures came from CRM 2D maps generated from the signal of the C=C stretching of LIN, at 1640 cm^–1^, and the C–H stretching signal, at 2920 cm^–1^ (Figure 10B).^62−65^ In fact, LIN and CIT drive the formation of core–shell capsules, with the polymer and the PRMs that synergistically form a shell around an aqueous core (as evident by tracking the O–H stretching band at 3400 cm^–1^), while no perfume was detected in water inside or outside the polymersomes (Figure 10B3). In these systems, the two PRMs seem to behave as a sort of cosurfactants for the polymer, favoring the formation of vesicle-like structures. Similar behavior was already proposed for fragrances with intermediate log *K*_ow_ values, which act as cosurfactants, interacting with polymeric micellar systems.^61^

Finally, the two perfumes with the higher log *K*_ow_ values (i.e., higher hydrophobicity), LIM and PIN, were found to drive the formation of what appear to be o/w emulsions: indeed, CLSM images show highly polydisperse dark aggregates against a red fluorescent background, suggesting that a polymer-rich aqueous phase surrounds and stabilizes a dispersion of polymer-less droplets. SANS measurements prove the micellar nature of the polymer-rich phase, as the scattering profiles of LIM and PIN samples are identical to the ones of 5% Soluplus (Figure S19). CRM analyses (Figure 10C), on the other hand, show that the two PRMs are only located inside the dark droplets seen in CLSM images. It is worth noting that the Raman signal of C=C and C–H stretching coming from Soluplus was much lower than the one coming from PRMs (see reference Raman spectra of pure materials in Supporting Information Figures S11–S17); this is why in Figure 10C1,10C2 Soluplus is not detectable outside the droplets, in the polymer-rich aqueous bulk phase. Moreover, quite interestingly, none of these hydrophobic perfume droplets appeared to coalesce upon contact with each other; this observation, corroborated by the long-term (>3 months) stability of the suspensions, suggests a remarkable elasticity of the interfacial polymer film that likely covers the droplet surface, leading to a particularly efficient stabilization mechanism against Ostwald ripening.

The above observations indicate that no clear relationship can be found between the log *K*_ow_ of the seven PRMs and the structures they form with Soluplus. In other words, contrary to what was expected, despite log *K*_ow_ is widely used to classify the nature of PRMs in perfume encapsulation studies, it proved not sufficient to predict the microstructure of PRM-based systems in the presence of an amphiphile polymer, like Soluplus.

More recently, a different and more advanced approach was used, employing the Hansen solubility parameters (HSPs) to predict and rationalize the phase behavior of water/PEG-*g*-PVAc/PRM ternary mixtures.^66^ This method allows a finer classification of PRMs, which are rated according to their affinity with the encapsulating polymer, also identifying the separate contributions to the total Hildebrand solubility parameter of dispersion, polar, and hydrogen bond forces. The HSP approach was also considered in the present case (see Section 14 in the SI for details), but no significant improvement was observed over the more traditional log *K*_ow_ method. The affinity of the seven PRMs was rated and correlated well with the PRM’s hydrophobicity scale (log *K*_ow_ method); nevertheless, this approach offered little help in understanding and predicting the different structures observed in the ternary systems.

However, if additional parameters are also considered, such as the presence of specific functional groups and molecular conformation, a clearer picture emerges. Four different structures were identified in the PRM/Soluplus/water systems: (i) swollen micelles (PE-based system); (ii) matrixlike particles (FLO- and CAR-based systems); (iii) vesicle-like particles (CIT- and LIN-based systems); and (iv) perfume emulsions stabilized by polymer micelles (PIN- and LIM-based systems). In fact, by combining the hydrophobicity, the presence of given functional groups, and the molecular conformation of the seven PRMs, these four structures can be justified and understood. More in detail, PE is sufficiently hydrophilic so that it is partitioned between the aqueous bulk and the micellar phases. The small fraction included in the micellar phase is easily solubilized into the slightly hydrophobic core of Soluplus micelles. FLO and CAR are characterized by a medium hydrophobicity, rather bulky molecules and the presence of carbonyl groups. This makes them very similar to the repeating monomeric units of Soluplus chains, and random PRMs/polymer mixing is particularly favored, resulting in the formation of matrix-like droplets. On the other hand, CIT and LIN are both fairly linear molecules terminated with hydroxyl groups. This gives them a slight amphiphilic character, which is reflected in their “cosurfactant” behavior, resulting in the formation of core–shell vesicle-like structures. Finally, LIM and PIN are the most hydrophobic among the considered PRMs; they are bulky molecules with no polar groups. Consequently, they do not mix either with water or with Soluplus, generating an emulsion-like structure stabilized by the presence of Soluplus micelles in the aqueous bulk phase.

## 4 Conclusions

Numerous commercial formulations in the market contain perfume, including home- and personal-care products, foodstuff, pesticides, antimicrobials, and others. Such industrial products containing perfumes or active molecules are in continuous need of improvement in relation to their efficiency, shelf life, and eco-compatibility.^15,67^ Amphiphilic polymers, forming aggregates like single-chain nanoparticles or micelles capable of solubilizing small hydrophobic molecules, are serious candidates for such applications. Here, Soluplus or PEG-*g*-(PVAc-co-PVCL), a biocompatible graft copolymer, was investigated in terms of its self-assembly properties in aqueous solutions and its ability to form supramolecular structures encapsulating different fragrances.

Polymer aqueous solutions were characterized up to 70% Soluplus (w/w) by means of SANS, rheology, and DSC analyses. It was found that, in the 1–15% concentration range, Soluplus micelles can be modeled as spherical particles with a fuzzy interface, having an average radius of about 22.4 nm, and interacting through a 2-Yukawa potential. These supramolecular aggregates were found to be highly hydrated, with a significant amount of water penetrating deep into the micelles’ core. On the other hand, SANS patterns from 20 to 55% Soluplus aqueous solutions were fitted using the Teubner–Strey model for bicontinuous structures, indicating relatively ordered micellar systems. By exploiting the SANS interaction peak position, it was shown that at least until Soluplus 45% w/w, micelles order themselves together, without disappearing or evolving into different structures, as observed extensively in literature for PNiPAM microgels.^37^

Soluplus was then tested as a potential encapsulating agent for seven fragrance molecules exhibiting not only different hydrophobic characters as expressed by their log *K*_ow_ value, but also different molecular characteristics (i.e., functional groups, molecular conformation, or bulkiness): 2-phenyl ethanol (PE), l-carvone (CAR), linalool (LIN), florhydral (FLO), β-citronellol (CIT), α-pinene (PIN), and *R*-limonene (LIM). It was found that the most hydrophilic of the PRMs used, PE, was solubilized in the polymeric micelles, causing a slight swelling and increasing of the micelle size as shown from SANS and ^1^H–^1^H NOESY analyses. All of the other PRMs led to the formation of micron-sized metastable structures. Combining CLSM and confocal Raman microscopy imaging, we found that FLO and CAR led to matrix-type capsules with the polymer and perfume homogeneously diffuse throughout the whole capsule volume. LIN and CIT drove the formation of core–shell vesicle-like microcapsules, remindful of polymersomes, where the perfume acts as a cosurfactant and is located in the shell of the dispersed particles together with the polymer. Finally, PIN and LIM formed a macroemulsion with perfume microdroplets stabilized by polymeric micelles in the aqueous medium.

The main result from these experiments demonstrated that the log *K*_ow_ parameter, widely used to determine the solubilization properties of hydrophobic molecules, is not sufficient to predict the structure and to understand the complex interaction that takes place when a perfume molecule interacts with a “complex” amphiphilic polymer, such as Soluplus. Previous studies have connected the hydrophobicity of fragrances and their log *K*_ow_ value with the capability to be solubilized in different compartments of such aggregates.^14,68^ Other studies have described the encapsulation of fragrances using the Hansen solubility parameters.^66,69^ Both approaches, however, at best are limited to rating the affinity of different PRM for the polymer, which is not enough to predict the behavior of such complex systems. In fact, nano- and microstructure of complex systems composed of water, an amphiphile polymer, and different fragrances can be efficiently predicted and described only if other parameters are also considered, such as the presence of specific functional groups and the molecular conformation of PRMs. These factors can generate specific and otherwise uneasily predictable interactions between small PRM molecules and polymer chains, which are reflected on a larger scale in the microstructure of the system. Accordingly, PRMs having different hydrophobicity but similar molecular structure and functional groups may interact in a similar way with a given polymer. Or, again, PRMs having very similar hydrophobicity but seemingly slightly different molecular structures may interact in a completely different way with a given polymer. This understanding will lead to the development of materials (polymers or other self-assembly molecules) able to encapsulate the desired range of PRMs. Achieving this will be of great scientific and industrial interest, as perfumes (but also active molecules) used in commercial formulations usually consist of a large number of PRMs that need to be effectively and stably encapsulated from production until consumer use of the final product.

## Supporting Information Available

HSQC, NOESY, and COSY NMR maps; tensiometry measurements; non-normalized SANS patterns; Kratky plot; Guinier plot; volume fraction calculations; modeling of SANS data; intermicellar distance vs reverse cubic root of Soluplus concentration; DSC thermograms in D_2_O; DSC calculations; volume fraction calculation; SANS fitting parameters and NOESY map of 5% Soluplus/1% 2-phenyl ethanol; Raman spectra of pure Soluplus and PRMs; Raman 2D maps of Soluplus/PRM capsules; SANS patterns of 5% Soluplus/1% α-pinene or *R*-limonene; and HSP approach (PDF)

## Notes

^∥^ No kinship exists among these authors.
