# Supplementary material for: Self-Assembly of Soluplus in Aqueous Solutions: Characterization and Prospectives on Perfume Encapsulation
Source: ACS Appl Mater Interfaces. 2022 Mar 21;14(12):14791–804. doi: 10.1021/acsami.2c01087 (PMC8972246; doi:10.1021/acsami.2c01087)
Supplement: Supplementary file 1 — am2c01087_si_001.pdf [file am2c01087_si_001.pdf]

Supporting Information

for

Self-assembly of Soluplus in aqueous solutions: characterization and prospectives on perfume encapsulation

Constantina Sofroniou,[1] Michele Baglioni,[1,a] Marianna Mamusa,[1] Claudio Resta,[1] James Doutch,[2] Johan Smets,[3] Piero Baglioni[1,a,]*

[1]Department of Chemistry "Ugo Schiff" and CSGI, University of Florence, Via della Lastruccia 3, Sesto Fiorentino, 50019 Florence, Italy

[2]Science and Technology Facilities Council, ISIS Neutron and Muon Source, Rutherford Appleton Laboratory, Didcot OX11 0QX, United Kingdom

[3]The Procter & Gamble Company, Temselaan 100, 1853 Strombeek Bever, Belgium

[a]No kinship exists among these authors.

1. NMR

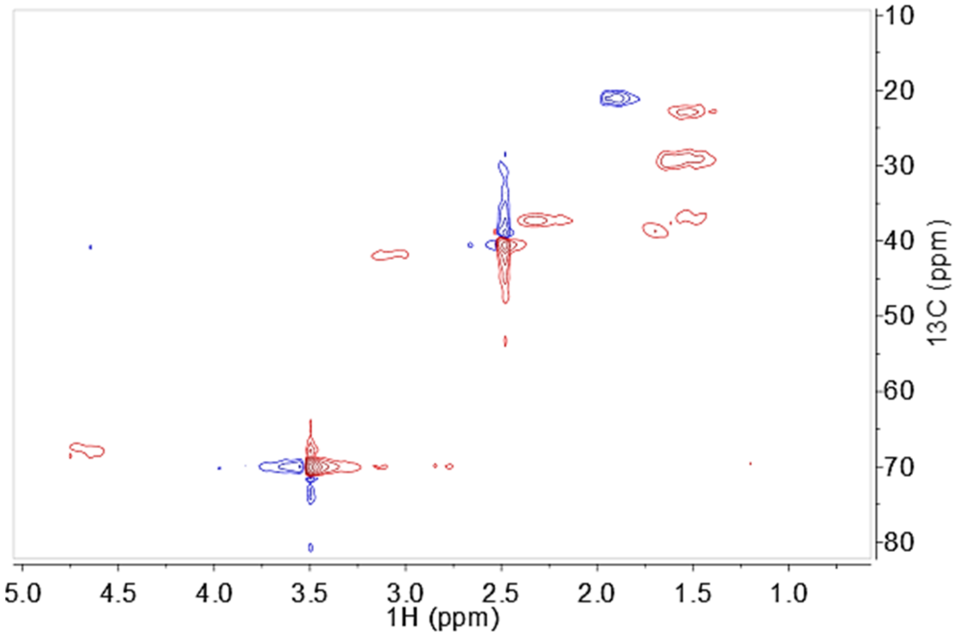

Figure S1. Soluplus 30 mg/ml in DMSO-d_6, HSQC map.

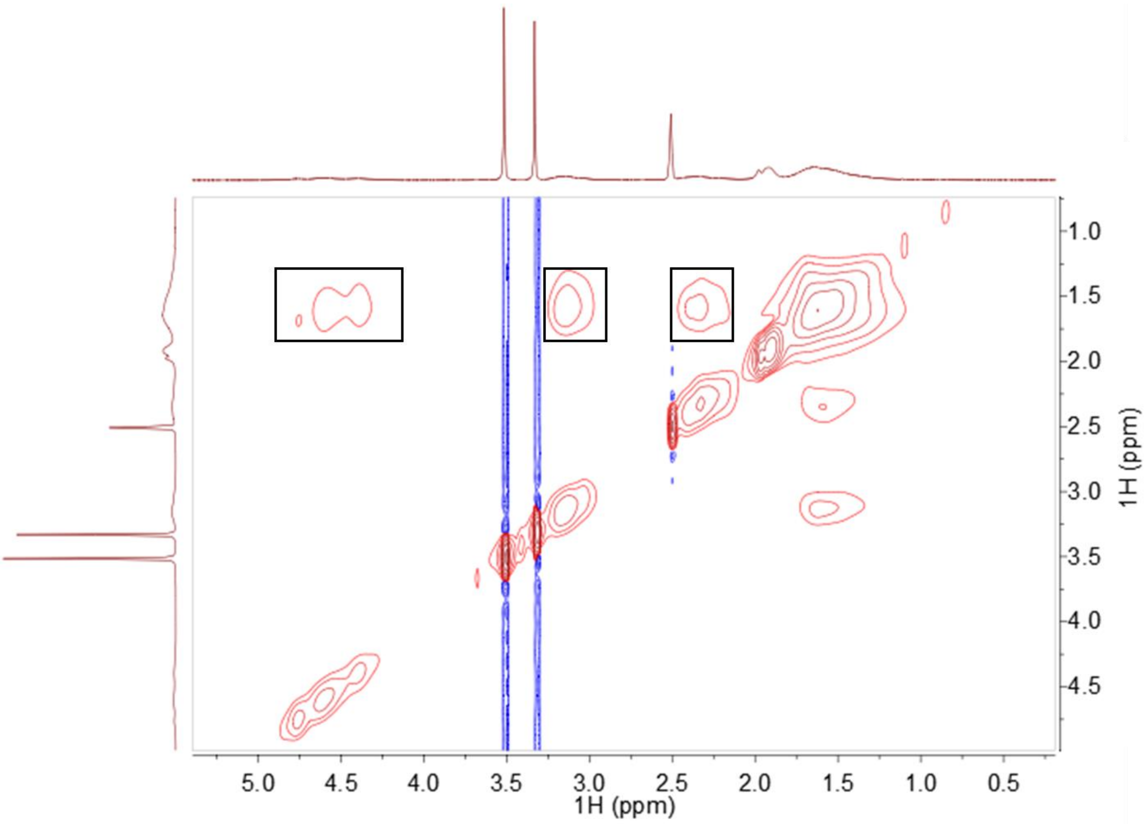

Figure S2. Soluplus 30 mg/ml in DMSO-d_6, NOESY map.

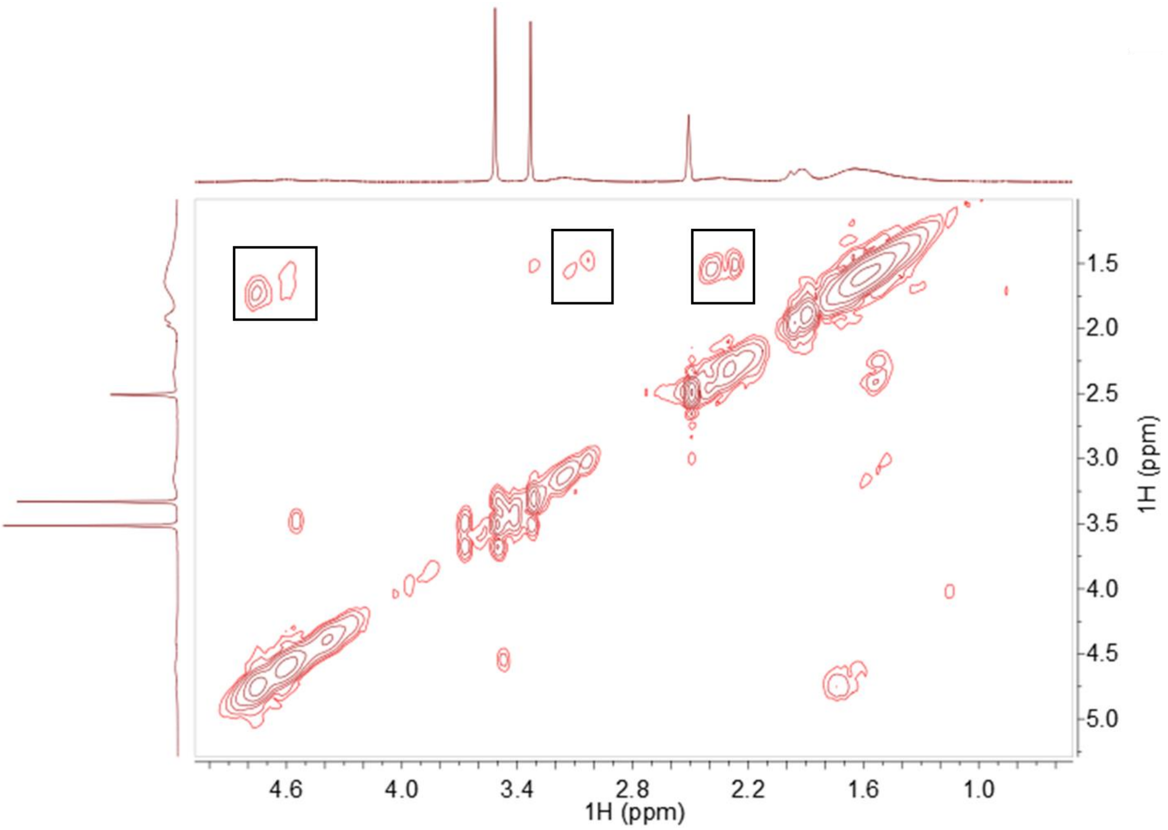

Figure S3. Soluplus 30 mg/ml in DMSO-d_6, COSY map.

2. Tensiometry

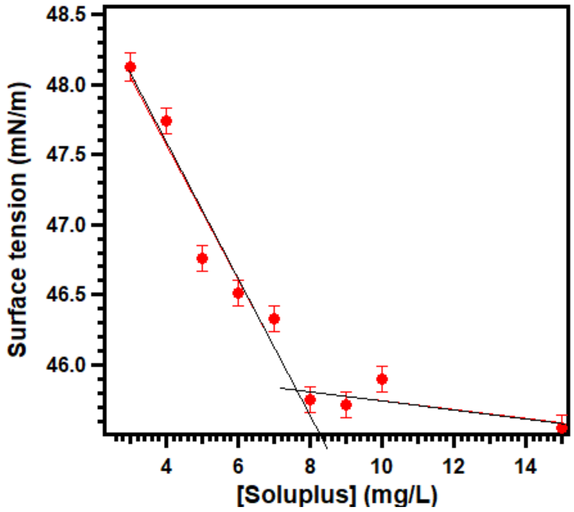

Figure S4. Surface tension vs Soluplus concentration.

3. SANS patterns

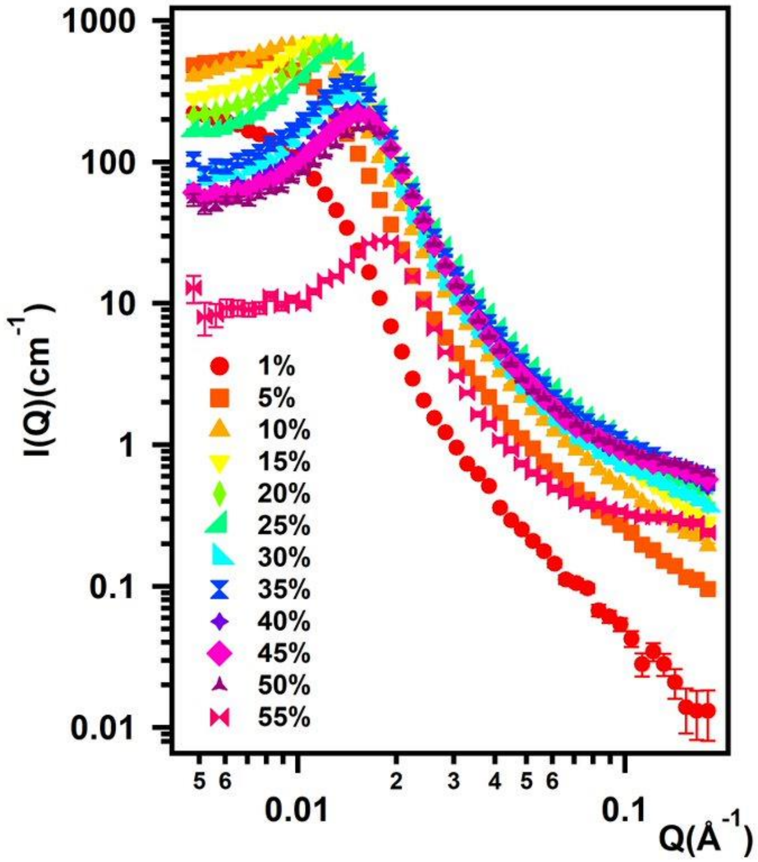

Figure S5. Non-normalized SANS curves for samples containing Soluplus in water; concentrations in the legend are %w/w.

4. SANS Kratky and Guinier plots

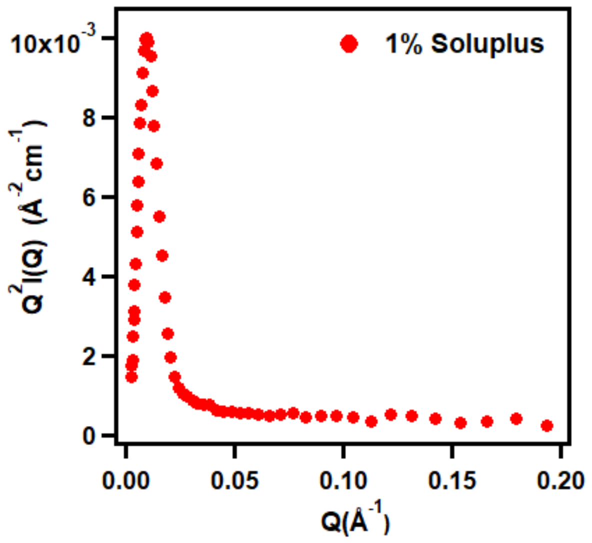

Figure S6. Kratky plot (Q^2*I(Q) vs. Q) for Soluplus 1% SANS pattern.

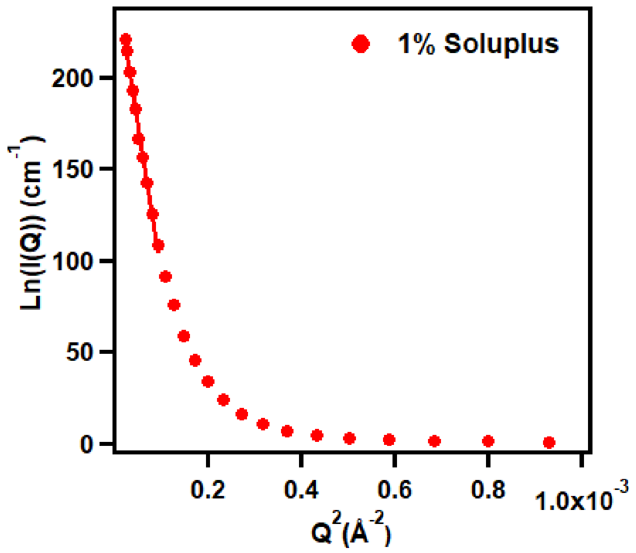

Figure S7. Guinier plot, Ln(I(Q) vs. Q^2, for Soluplus 1% SANS pattern.

5. Volume fractions

The theoretical polymer solution volume fractions (Φ) were calculated assuming the additivity of volumes and densities for the components of the polymer and the solvent by using the expression:

$$\Phi = \frac{f_\omega}{f_\omega + (1 - f_\omega)\frac{\rho_p}{\rho_s}} \qquad \text{Eq. S1}$$

Where f_ω is the weight fraction, ρ_p is the polymer density equal to 1.08 g/cm^3 [1] and ρ_s the density of water at 25 ºC that equals to 0.997 g/cm^3. The volume fractions obtained with this calculation are summarized in **Table S1**.

Table S1. Conversion of Soluplus volume fractions from wight fractions by using Eq. S1.

[Soluplus] % w/w	%Volume fraction
1	0.9
5	4.6
10	9.3
15	14.0
20	18.8
25	23.5
30	28.3
35	33.2
40	38.1
45	43.0
50	48.0
55	53.0

6. Modeling of SANS data

Fuzzy sphere model

The SANS data of 1%-15% Soluplus in D_2O were fitted using the form factor of a fuzzy sphere combined with a double Yukawa (2Y) interaction potential. The scattering intensity of a fuzzy sphere can be approximated as:[2,3]

$$I(Q) = \frac{\Phi}{V}(\Delta p)^2 P(Q) S(Q) + I_{fluct}(Q) + B \qquad \text{Eq. S2}$$

Where Δ_p is the scattering length density (SLD) difference between the sphere and the solvent, Φ is the volume fraction of particles and V the sphere volume. The form factor, P(Q) is given by:

$$P(Q) = \left[\frac{3(\sin QR_c - QR_c \cos QR_c)}{(QR_c)^3} \exp\left(\frac{-(\sigma Q)^2}{2} \right) \right]^2 \qquad \text{Eq. S3}$$

Where R_c is the radius of the core and σ the width of the fuzzy sphere surface. R_c represents the radius where the density of the core decreases to ½. The particle size distribution is taken into account via a Gaussian function:

$$D(R_c, \langle R_c \rangle, \sigma_{pol}) = \frac{1}{\sqrt{2\pi \sigma_{pol}{}^2 \langle R_c \rangle^2}} \exp\left(-\frac{(R_c - \langle R_c \rangle)^2}{2\sigma_{pol}{}^2 \langle R_c \rangle^2} \right) \qquad \text{Eq. S4}$$

Where $\langle R_c \rangle$ is the average core radius and σ_{pol} the particle polydispersity. Moreover, I_{fluct} is a Lorentzian term that accounts for the inhomogeneities of the polymer chains at high Q values and they can be described as:

$$I_{fluct}(Q) = \frac{I_{fluct}(0)}{1 + (\xi Q)^2} \qquad \text{Eq. S5}$$

Where $I_{fluct}(0)$ is the intensity at Q=0 and ξ is the correlation length of the fluctuations, related with the blob size. The inter-particle interactions were described using a 2Y potential represented with the equation:[4]

$$\frac{V(r)}{k_B T} = \begin{cases} \infty, (0 < r < 1) \\ -K_1 \frac{\exp(-Z_1(r-1))}{r} - K_2 \frac{\exp(-Z_2(r-1))}{r}, r > 1 \end{cases} \qquad \text{Eq. S6}$$

and the mean spherical approximation (MSA) closure

$$\begin{cases} h(r) = -1, (0 < r < 1) \\ c(r) = \frac{-V(r)}{k_B T} = K_1 \frac{\exp(-Z_1(r-1))}{r} + K_2 \frac{\exp(-Z_2(r-1))}{r}, r > 1 \end{cases} \qquad \text{Eq. S7}$$

Where r is the interparticle separation normalized by the core diameter, Z_1 and Z_2 are the apparent charges inversely proportional to the interaction ranges and K_1 and K_2 are scaling factors that account for the strength of the two potentials. The 2Y potential can take the form of a short-range attraction and a long-range repulsion if $Z_1 > Z_2$, or that of a short-range repulsion and a long-range attraction if

$Z_1<Z_2$. If the scale factor K_2 is negative, the first term of Eq. S6 represents the attractive component and the second term represents the repulsive component of the potential.

The theoretical neutron SLD values of the pure components were calculated for comparison reasons using the equation and can be seen in Table S2:

$$SLD = \frac{N_A \rho}{\mathrm{M}} \sum b_i \qquad \text{Eq. S8}$$

where N_A is Avogadro's number, ρ is the bulk density and M the molecular weight of the monomeric block respectively, and b_i is the coherent scattering length of atom *i*.

Teubner-Strey model

The scattering intensity of a two-phased system described by the TS model can be characterized by the form:[5]

$$I(Q) = \frac{1}{a_2 + c_1 Q^2 + c_2 Q^4} + B \qquad \text{Eq. S9}$$

Where B is the background and parameters α_2, c_1 and c_2 are coefficients that can be defined in terms of two length scales, a correlation length (ξ) and periodicity (d) that is a measure of the repeated distance between a water-rich and surfactant-rich domain:

$$a_2 = \left[1 + \left(\frac{2\pi\xi}{d}\right)^2\right]^2 \qquad \text{Eq. S10}$$

$$c_1 = -2\xi^2 \left(\frac{2\pi\xi}{d}\right)^2 + 2\xi^2 \qquad \text{Eq. S11}$$

$$c_2 = \xi^4 \qquad \text{Eq. S12}$$

Thus, ξ and d are obtained by:

$$\xi = \left[\frac{1}{2}\left(\frac{a_2}{c_2}\right)^{1/2} - \frac{1}{4}\frac{c_1}{c_2}\right]^{-1/2} \qquad \text{Eq. S13}$$

$$d = 2\pi \left[\frac{1}{2}\left(\frac{a_2}{c_2}\right)^{1/2} - \frac{1}{4}\frac{c_1}{c_2}\right]^{-1/2} \qquad \text{Eq. S14}$$

Knowing ξ and d, one can define the amphiphilicity factor (f_α) which characterizes the ordering of the system:

$$f_a = \frac{c_1}{(4 a_2 c_2)^{1/2}} \qquad \text{Eq. S15}$$

Additionally, a Lorentzian term was included in the fitting procedure that accounts for the enhanced density fluctuations of the polymer chains are the micellar surface as the Q increases. Thus, the final fitting equation takes the form of:

$$I(Q) = \frac{\Phi(\Delta\rho)^2 (2\pi / \xi) c_2}{a_2 + c_1 Q^2 + c_2 Q^4} + \frac{I(0)}{1 + (\xi_{Lor} Q)^2} + B$$

Eq. S16

Where except of ξ and d, the scattering intensity is a function of the volume fraction (Φ) and contrast (Δρ). I(0) is the intensity at Q=0 and ξ_{Lor} the correlation length of the Lorentzian term.

Table S2. Neutron scattering length densities (SLDs) as calculated for the three repeated units of Soluplus, D_2O and 2-phenyl ethanol (PE).

Monomeric unit	Neutron SLD, ρ (Å^{-2})
PEG	6.7 10^{-7}
PVAc	9.89 10^{-7}
PVCL	8.78 10^{-7}
D_2O	6.4 10^{-6}
PE	1.09 10^{-6}

7. Intermicellar distance vs. Soluplus concentration

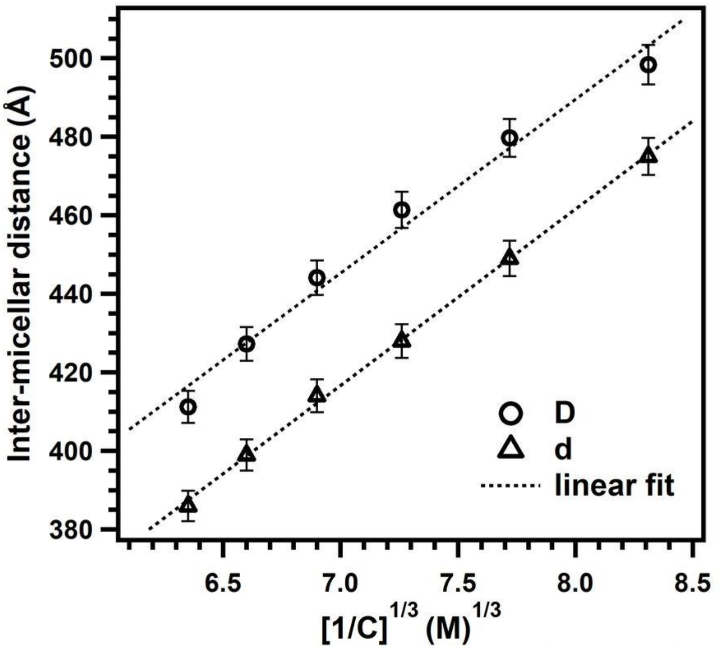

Figure S8. Intermicellar distance, D (derived from $2\pi/Q_{max}$) or d (obtained after fitting with the TS model) vs. the reverse cubic root of the polymer concentration. R^2=0.98, slope = 42 ± 2 for D; R^2=0.99, slope = 43 ± 1 for d.

8. Differential Scanning Calorimetry

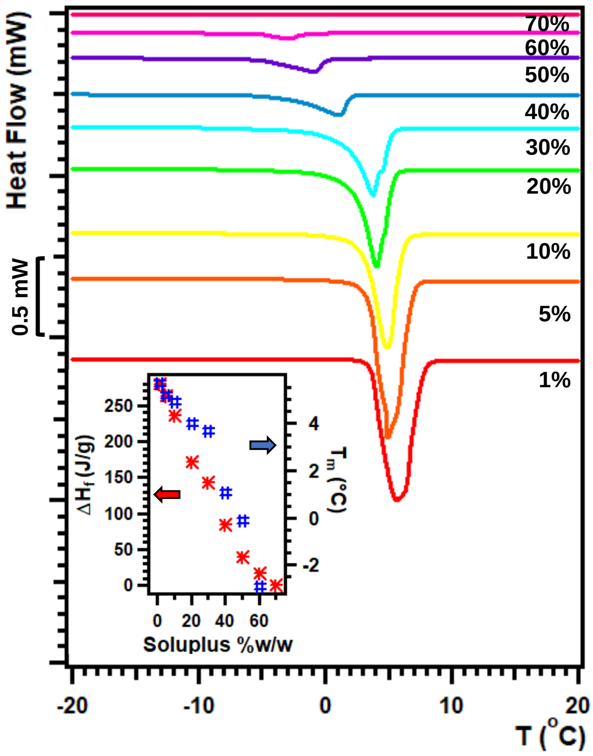

Figure S9. DSC thermogram for Soluplus samples in D_2O. Inset: enthalpies of fusion (ΔH_f, J/g) and melting temperatures (T_m, °C) as obtained after integration of each peak in the DSC thermogram.

9. Calculations from DSC data

Water molecules per EO unit

For Soluplus 70%, no endothermic peak appears in the DSC thermogram (**Figure 6** of the main text or **Figure S9** of the SI) indicating that all the water is bound to Soluplus. Initially, in the sample the water content was 30%, which is all bound to Soluplus. The mass of this bound water is is 0.3 g = 0.0167 mol. In the same way, the initial mass of Soluplus in 1 g sample was 0.7 g = 6.09 10^{-6} mol. In this way we obtain 2738 water molecules per Soluplus chain. Considering that EO units are the 13% of a Soluplus chain mass, this equals to 2.48 10^{-20} g as mentioned above, by dividing with the mass of a single EO unit (44 g/mol / N_{AV}) we obtain 340 EO units per Soluplus chain. By dividing the water molecules bound to a chain, with the 340 EO units, this gives 8 H_2O molecules per EO unit.

N_{agg} for Soluplus 1%

For Soluplus 1% w/w by integration of the endothermic peak around 0 °C of **Figure 6**, we obtain $\Delta H_{f,sample}$ = 280.2 J/g. By using Equation 1 from the main text:

$$FWC = \frac{\Delta H_{f,sample} . m_{sample}}{\Delta H_{f,water} . m_{water}} \times 100$$

this leads to an FWC of 92.3% and the remaining 7.8% is the water bound to the micelles. Initially, in the sample the water content was 99%, from which 7.8% is bound to the micelles, and considering one gram of sample, the mass of bound water is 0.076 g = 0.0038 mol. In the same way, the initial mass of Soluplus in 1 g sample was 0.01 g = 8.69 10^{-8} mol. So, there are 4.38 10^{4} water molecules per Soluplus chain.

The volumes of the PEG backbone and the VAc-VCL graft of Soluplus were calculated theoretically by using the density expression d = V/m where d is the density of the backbone or the graft, V the volume and m the mass. The density and molar mass for each component are summarized in **Table S3**.

From the NMR results, the mass ratio between the three components was found to be 13% EO, 34% VAc and 53% VCL. One Soluplus chain weights 1.91 10^{-19} g, from which 2.48 10^{-20} g is PEG and the rest 1.66 10^{-19} is the graft. For the density of the VAc-VCL graft, the average of the density of the two components was used. The results gave: $V_{PLC-PVAc}$ = 166170 Å^3 and V_{PEG}= 22162 Å^3.

The total volume of each chain is: $V_{chain} = V_{PEG} + V_{PCL-PVAc} + V_{H2O}$ = 1.5 10^{6} Å^3

Where V_{D2O} was calculated by multiplying the number of water molecules per Soluplus chain with the volume of a water molecule that equals 30 Å^3. The volume of each micelle that can be calculated using the micelle radius obtained from the SANS fittings and the following formula for a sphere's volume:

$$V_{micelle} = \frac{4}{3}\pi R_{SANS}{}^{3} \quad \text{Eq. S17}$$

By combining the micelle volume with that of each chain we can estimate the aggregation number according to the equation:

$$V_{micelle} = V_{chain} N_{agg} \quad \text{Eq. S18}$$

Where for 1% Soluplus in D_2O and R_{SANS} = 223 Å, the obtained N_{agg} is equal to 30.5.

Table S3. Density and molar mass for each monomeric unit of Soluplus.

Monomeric unit	Molar mass (g/mol)	Density (g/cm^3)
EO	44.05	1.11
VAc	86.09	0.934
VCL	139.19	1.029

10. SANS pattern fitting and NOESY map of 5% Soluplus / 1% 2-phenyl ethanol

Table S4. Structural parameters obtained by fitting the 5% Soluplus / 1% 2-phenyl ethanol in D_2O SANS curve (Figure 8 of main text) to Eq. 4. The parameters for which no error is given were kept fixed during the fitting procedure.

Parameter	Value
Volume fraction	0.24
Core radius, Rcore (Å)	222 ± 4
Core polydispersity	0.27
Fuzziness, σ (Å)	25
Core SLD (Å-2)	4.0 10-6
Solvent SLD (Å-2)	6.4 10-6
Lorentzian scale	5.3
Lorentzian length (Å)	50
Attraction strength	4.12 ± 0.05
Attraction range	28.0 ± 0.1
Repulsion strength	-1.00 ± 0.01
Repulsion range	1.50 ± 0.01

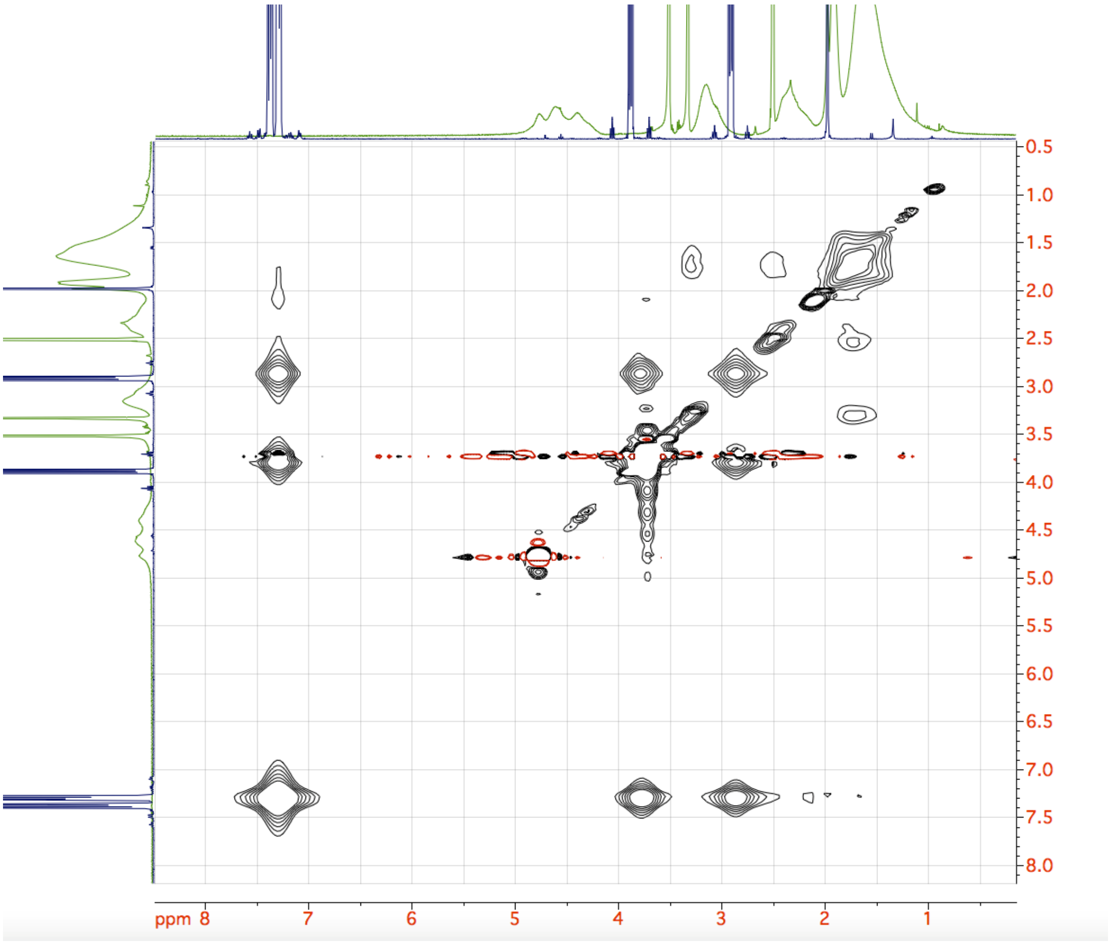

Figure S10. NOESY map of 5% Soluplus / 1% 2-phenyl ethanol in D_2O. On both axes ^{1}HNMR spectra of 2-phenylethanol in $CDCl_3$ and Soluplus in DMSO-d_6 are respectively depicted in blue and green. Such spectra are use as indicators of specific resonances of the two systems.

11. Raman spectra of pure compounds

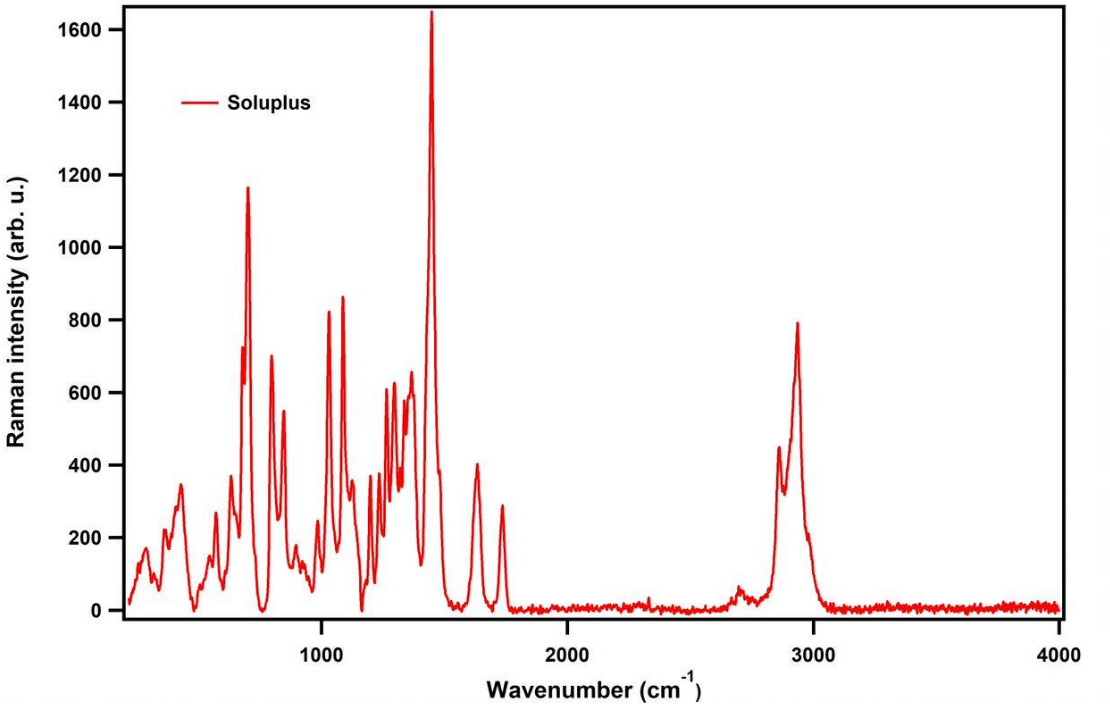

Figure S11. Reference Raman spectra of pure Soluplus after excitation wavelength 785 nm (wavenumber range from 300 to 3700 cm^{-1} using the extended range mode).

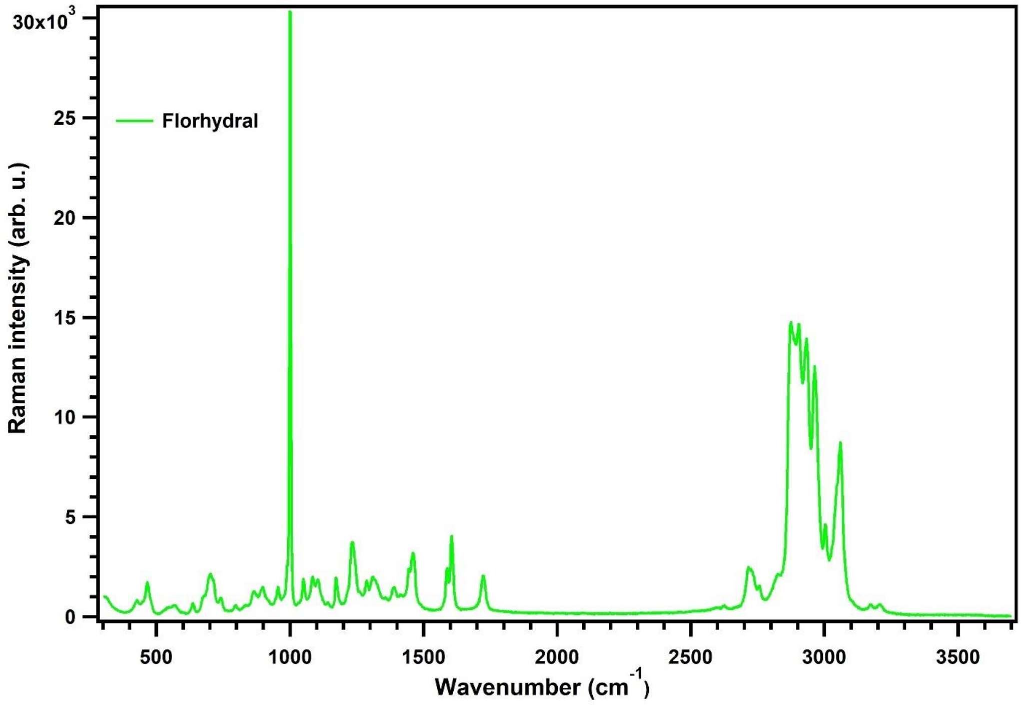

Figure S12. Reference Raman spectra of pure florhydral after excitation wavelength 532 nm (wavenumber range from 300 to 3700 cm^{-1} using the extended range mode).

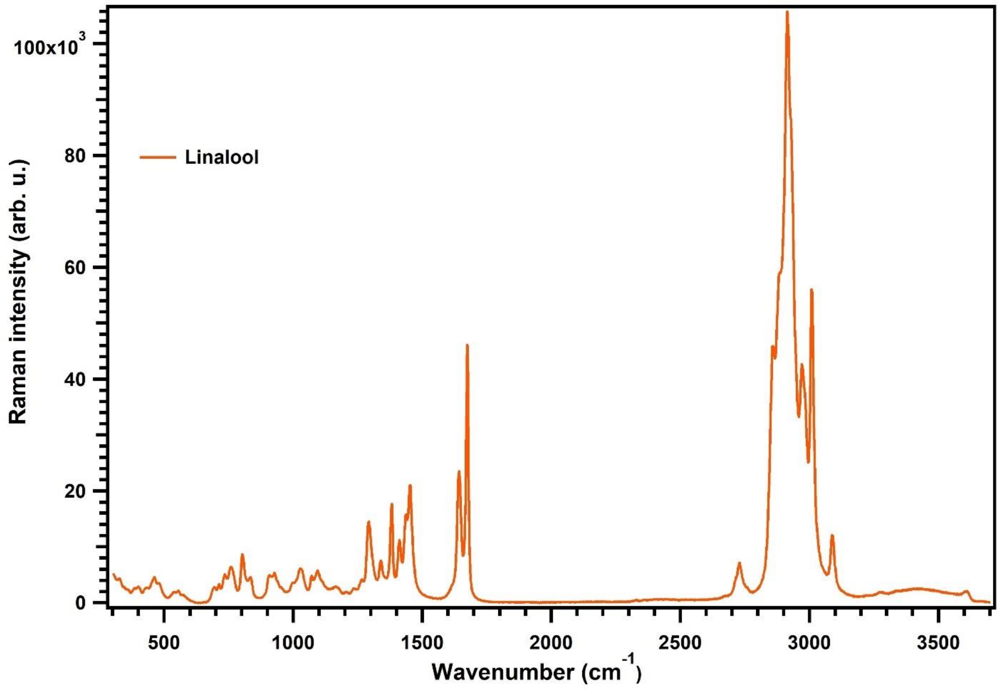

Figure S13. Reference Raman spectra of pure linalool after excitation wavelength 532 nm (wavenumber range from 300 to 3700 cm^{-1} using the extended range mode).

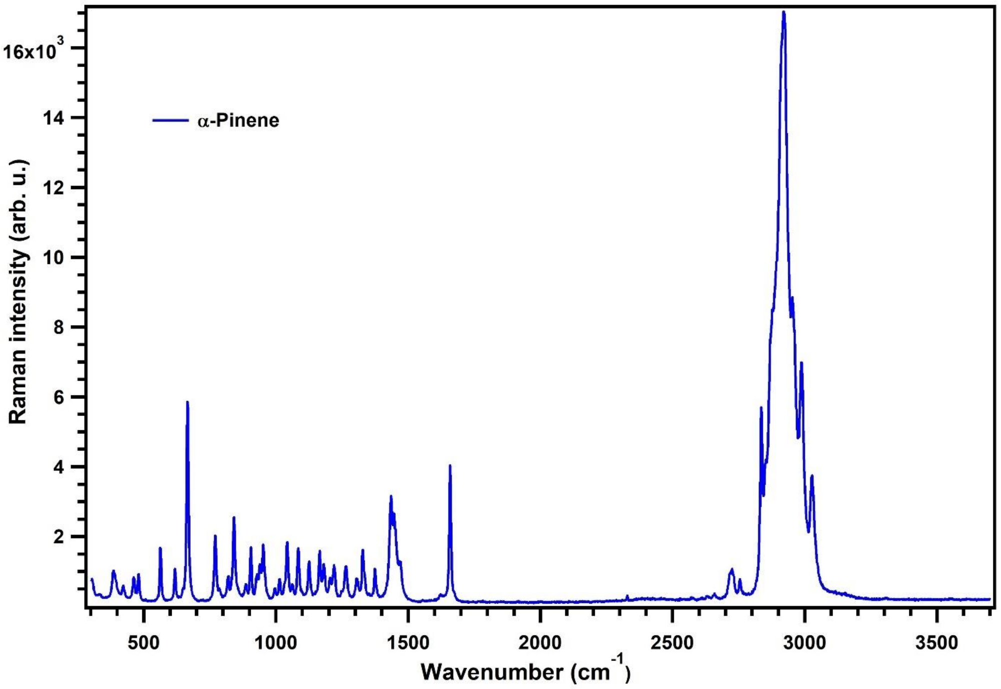

Figure S14. Reference Raman spectra of pure α-pinene after excitation wavelength 532 nm (wavenumber range from 300 to 3700 cm^{-1} using the extended range mode).

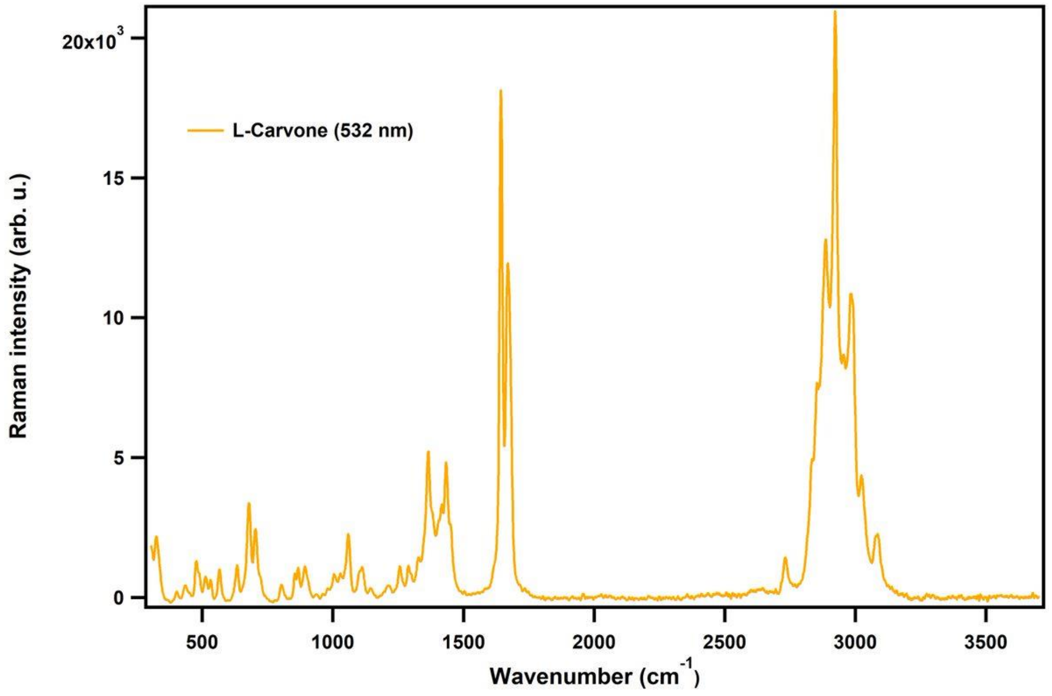

Figure S15. Reference Raman spectra of pure L-carvone after excitation wavelength 532 nm (wavenumber range from 300 to 3700 cm^{-1} using the extended range mode).

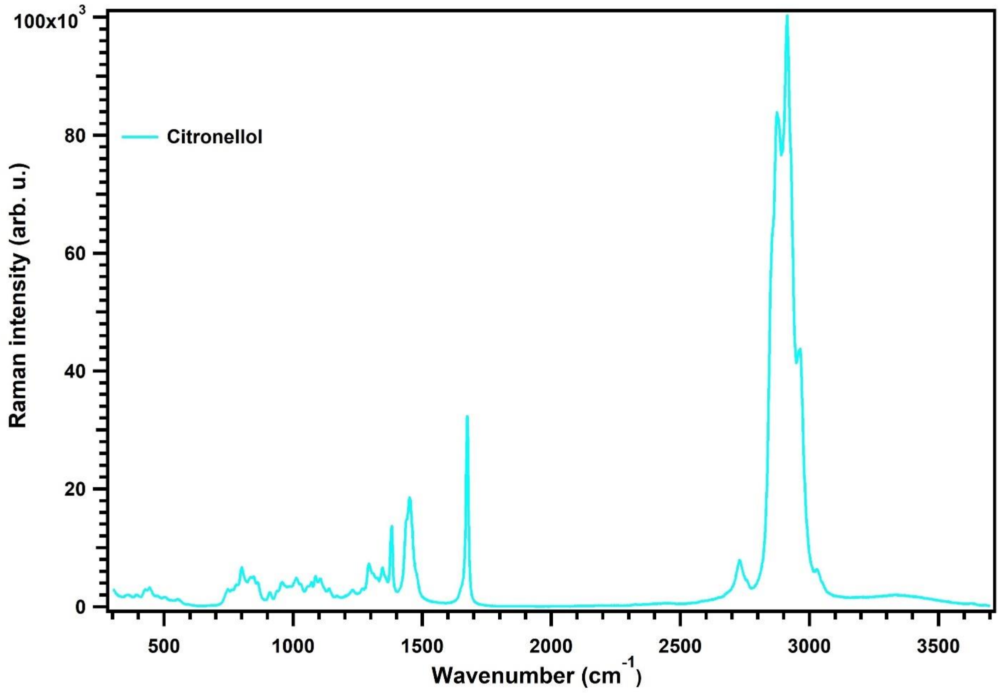

Figure S16. Reference Raman spectra of pure β-citronellol after excitation wavelength 532 nm (wavenumber range from 300 to 3700 cm^{-1} using the extended range mode).

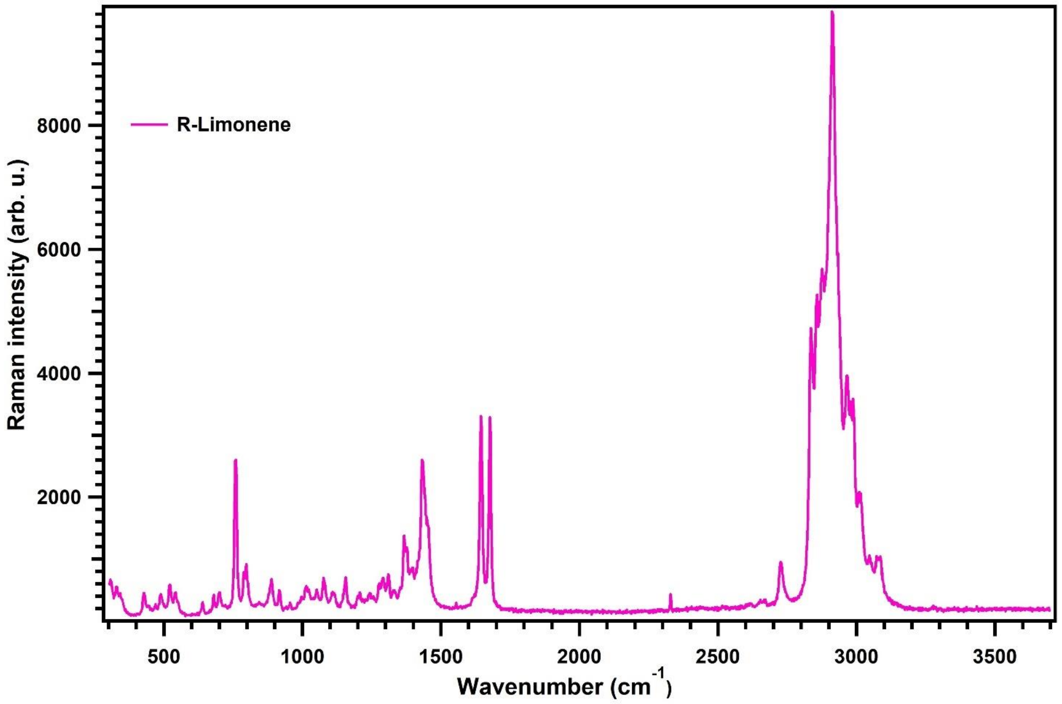

Figure S17. Reference Raman spectra of pure R-limonene after excitation wavelength 532 nm (wavenumber range from 300 to 3700 cm^{-1} using the extended range mode).

12. 2D Raman mapping

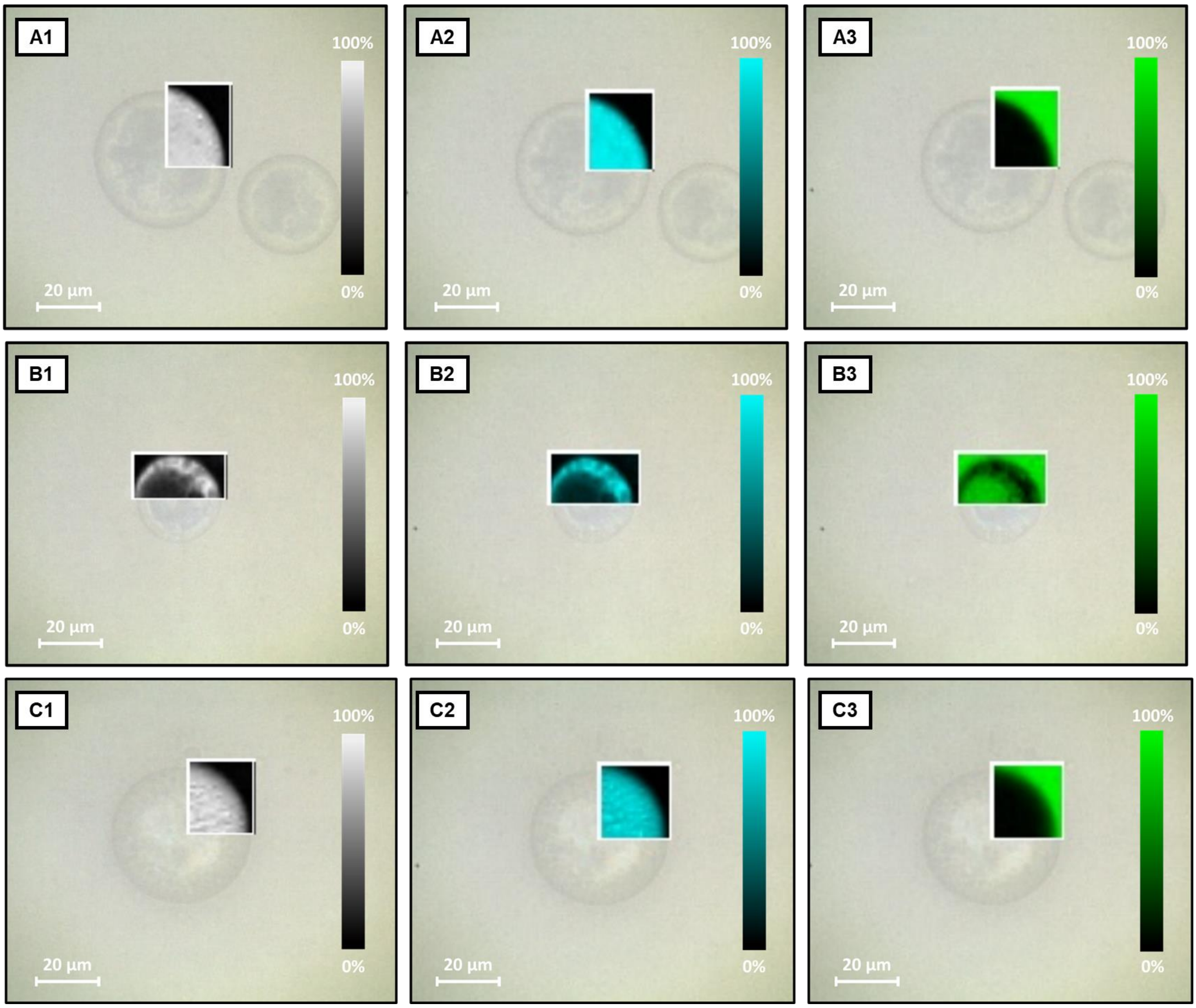

Figure S18. Raman 2D mapping (LWD 50× objective) of 94% w/w water, 5% polymer and 1% of each of the perfumes: A) L-carvone, B) β-citronellol, C) R-limonene. The different colors represent tracking of the different Raman signals. White signal: tracking at 1640 cm^{-1} (C=C stretching band). Blue signal: Tracking at 2920 cm^{-1} (C-H stretching band). Green signal: Tracking at 3400 cm^{-1} (O-H stretching band).

13. SANS patterns of 5% Soluplus / 1% α-Pinene or R-Limonene

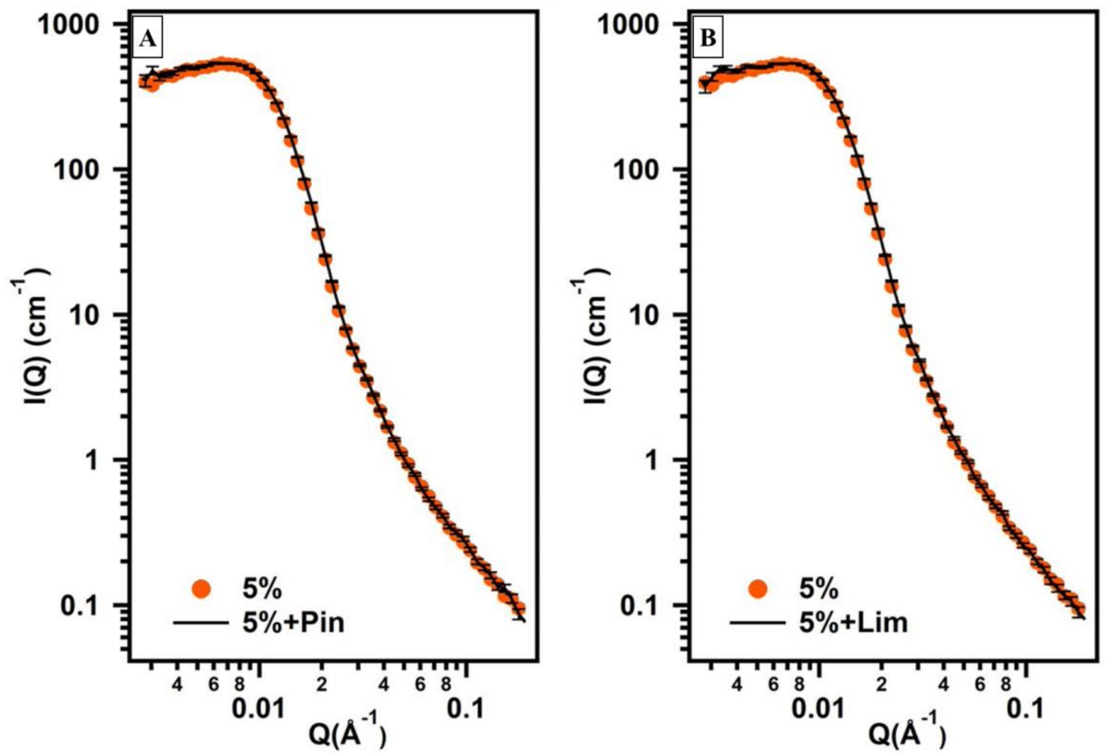

Figure S19. SANS patterns obtained for the samples containing 5% Soluplus and 1% A) α-Pinene or B) R-Limonene in D_2O. Markers represent experimental points of 5% Soluplus aqueous solution in the absence of PRM and solid lines represents the experimental data of the two samples.

14. HSPs Approach

The HSP sphere was built using the HSPiP (Hansen Solubility Parameters in Practice) software, © 2008-20 Steven Abbott and Hiroshi Yamamoto, www.hansen-solubility.com, and can be seen in **Figure S20**. After creating the solubility sphere, the distance (D) between the polymer (or the solute of interest) and a solvent in the solubility space can be calculated:

$$(D)^2 = 4(\delta_{D2} - \delta_{D1})^2 + (\delta_{P2} - \delta_{P1})^2 + (\delta_{H2} - \delta_{H1})^2 \quad \text{Eq. S19}$$

The RED (from Relative Energy Difference) number for a given solvent is then obtained as:

$$RED = D / R_0 \quad \text{Eq. S20}$$

where R_0 is the sphere radius. The RED is defined in such a way that is < 1 for good solvents (inside the HSP sphere), and > 1 for bad solvents (outside the HSP sphere).[6,7]

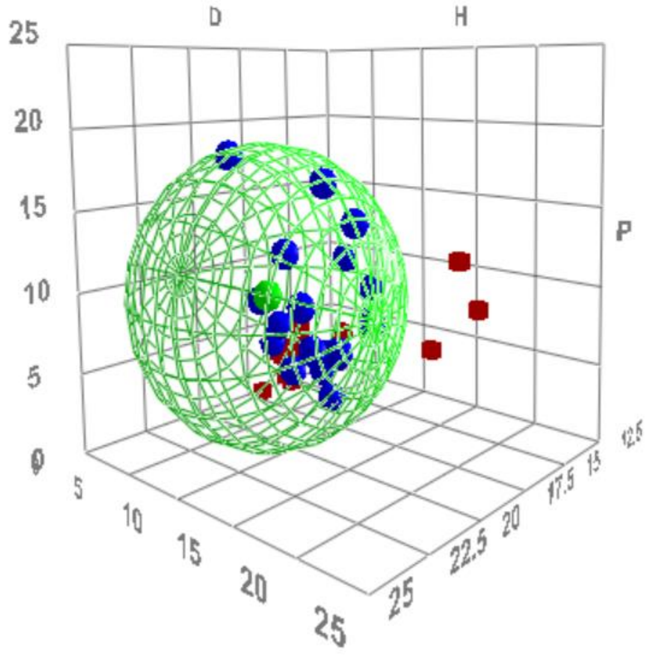

Figure S20. Hansen solubility sphere for Soluplus as calculated from the HSPiP software after solubility experiments using a series of solvents, including the seven PRMs. The three axes correspond to the three Hansen solubility parameters: D = Dispersion forces parameter, P = Polar interactions parameter, H = Hydrogen bonding parameter. The green sphere represents the surface of the calculated HSP sphere, the small blue spheres represent good solvents; the red cubes represent bad solvents; the small green sphere in the centre of the HSP sphere represents the coordinates of Soluplus's HSPs.

In this case, the obtained HSPs for Soluplus (centre of the Sphere) are: $\delta_D = 20.1$, $\delta_P = 9.5$, $\delta_H = 8.1$. The HSP parameters of the seven PRMs selected for the present study are reported in **Table S5**.

Table S5. PRMs tested as solvents for Soluplus solubilization studies, their HSP values (as obtained from the HSPiP software), Hildebrand parameter (δ) obtained from $\delta^2 = \delta_D^2 + \delta_H^2 + \delta P^2$ and RED value obtained using Eq. S20.

PRM	δ_D MPa$^{1/2}$	δ_P MPa$^{1/2}$	δ_H MPa$^{1/2}$	δ	RED	log K_{ow}
Soluplus	20.1	9.5	8.1	23.66157	-	-
2-Phenyl Ethanol	18.3	5.6	11.2	22.17408	0.650	1.36
L-Carvone	17.5	5.8	3.7	18.80372	0.814	2.74
Citronellol	16.5	4	7.9	18.72592	0.955	3.30
Linalool	16.8	2.9	6.9	18.39185	0.990	2.97
Florhydral	17.3	4.3	2.5	18.00083	0.994	3.02
R-Limonene	17.2	1.8	4.3	17.82049	1.087	4.57
α-Pinene	16.9	1.8	3.1	17.27599	1.174	4.44

The Hildebrand parameter (δ) theory states that two substances are effectively miscible if they have similar δ values. Table S5 shows that δ follows the sequence PE, CAR, CIT, LIN, FLO, LIM, PIN, with PE being the closest to the δ value of Soluplus. This sequence follows the exact same order of the RED values obtained after creating the HSP sphere, with PE being the closest to the centre of the sphere (Soluplus coordinates) and PIN being the furthest. So, other than evidently showing that that PE, CAR, LIN, FLO and CIT have higher affinity with the polymer, while PIN and LIM are bad solvents for it, no apparent correlation is observed between the HSP parameters (δ or RED value) and the different microstructures of water/PRM/Soluplus ternary systems. Actually, looking at the last column of Table S5, where log K_{ow} values of the seven PRMs are reported, it is worth noting that HSPs and log K_{ow} approaches provide similar (and coherent) information, i.e. five PRMs have higher affinity for Soluplus, while the two most hydrophobic ones have poor affinity for the polymer. Indeed, the HSP approach has the advantage of clearly showing the threshold (RED = 1) between good and bad solvents, but other than that no significant improvement is inferred over the more log K_{ow} traditional approach. Also, taking a closer look at the individual HSP parameters of the four intermediate PRMs, CAR, LIN, FLO and CIT, it does not provide additional helpful information.

LIN and CIT have higher δ_H values (6.9 and 7.9 respectively) compared to CAR and FLO (3.7 and 2.5 respectively). LIN and CIT are thus characterized by a stronger hydrogen bonding ability, as expected in view of the hydroxyl groups present in their molecules. This should add to their cosurfactant behavior, by locating the molecules in such position for the formation of vesicle-like structures, where the hydrogen-bonding is favoured with water molecules inside and outside of the capsules. δ_P values of the four PRMs are closer to each other, specifically 5.8 and 4.3 for CAR and FLO respectively, and 4.0 and 2.9 for CIT and LIN respectively. Similarly, for the δ_D values of the four PRMs, the value is 17.5 and 17.3 for CAR and FLO respectively, and 16.5 and 16.8 for CIT and LIN respectively. Thus, further interpretation using these parameters is risky. Additionally, the accuracy of the δ_D, δ_H, and δ_P values obtained from the software needs to be considered. One example is the δ_H value of PIN. The HSPiP gives a δ_H value of 3.1 for PIN. On the other hand, in a paper by Hofmeister et al. a value of 0 is given for the δ_H of PIN.[8]

In conclusion, the HSP theory can give more detailed and refined information on the polymer – PRM affinity than the traditional approach based on log K_{ow}; however, present study shows that both these approaches are not sufficient to effectively predict, justify and understand the different microstructures observed.

References

(1) Altamimi, M. A.; Neau, S. H. Investigation of the in Vitro Performance Difference of Drug-Soluplus® and Drug-PEG 6000 Dispersions When Prepared Using Spray Drying or Lyophilization. Saudi Pharm. J. 2017, 25 (3), 419–439. https://doi.org/10.1016/j.jsps.2016.09.013.

(2) Stieger, M.; Pedersen, J. S.; Lindner, P.; Richtering, W. Are Thermoresponsive Microgels Model Systems for Concentrated Colloidal Suspensions? A Rheology and Small-Angle Neutron Scattering Study. Langmuir 2004, 20 (17), 7283–7292. https://doi.org/10.1021/la049518x.

(3) Stieger, M.; Pedersen, J. S.; Lindner, P. Small-Angle Neutron Scattering Study of Structural Changes in Temperature Sensitive Microgel Colloids Sensitive Microgel Colloids. J. Chem. Phys. 2004, 120 (January 2004), 6197–6206. https://doi.org/10.1063/1.1665752.

(4) Chen, S. H.; Broccio, M.; Liu, Y.; Fratini, E.; Baglioni, P. The Two-Yukawa Model and Its Applications: The Cases of Charged Proteins and Copolymer Micellar Solutions. J. Appl. Crystallogr. 2007, 40 (SUPPL. 1), 321–326. https://doi.org/10.1107/S0021889807006723.

(5) Teubner, M.; Strey, R. Origin of the Scattering Peak in Microemulsions. J. Chem. Phys. 1987, 87, 3195–3200. https://doi.org/10.1063/1.453006.

(6) Hansen, C. M. Hansen Solubility Parameters: A User’s Handbook; CRC Press, Taylor & Francis Group, 2007.

(7) Hansen, C. M. The Three Dimensional Solubility Parameter and Solvent Diffusion Coefficient. Their Importance in Surface Coating Formulation, Danish Technical Press, Copenhagen, 1967.

(8) Hofmeister, I.; Landfester, K.; Taden, A. Controlled Formation of Polymer Nanocapsules with High Diffusion-Barrier Properties and Prediction of Encapsulation Efficiency. Angew. Chem. - Int. Ed. 2015, 54 (1), 327–330. https://doi.org/10.1002/anie.201408393.
